# Opioid system modulation with buprenorphine/samidorphan combination for major depressive disorder: two randomized controlled studies

**DOI:** 10.1038/s41380-018-0284-1

**Published:** 2018-10-29

**Authors:** Maurizio Fava, Michael E. Thase, Madhukar H. Trivedi, Elliot Ehrich, William F. Martin, Asli Memisoglu, Narinder Nangia, Arielle D. Stanford, Miao Yu, Sanjeev Pathak

**Affiliations:** 1 Massachusetts General Hospital Clinical Trials Network and Institute (CTNI), Harvard Medical School, Boston, MA USA; 2 University of Pennsylvania Perelman School of Medicine and the Corporal Michael Crescenz Veterans Affairs Medical Center, Philadelphia, PA USA; 3 University of Texas Southwestern Medical Center, Dallas, TX USA; 4 Alkermes, Inc., Waltham, MA USA

**Keywords:** Depression, Neuroscience

## Abstract

The endogenous opioid system is thought to play an important role in the regulation of mood. Buprenorphine/samidorphan (BUP/SAM) combination is an investigational opioid system modulator for adjunctive treatment of major depressive disorder (MDD). To confirm results from early studies, we report the efficacy and safety of BUP/SAM as adjunctive treatment in patients with MDD and an inadequate response to antidepressant therapy (ADT) in FORWARD-4 and FORWARD-5: two phase 3, randomized, double-blind, placebo-controlled studies that utilized the same sequential parallel-comparison design. Efficacy was measured using the Montgomery–Åsberg Depression Rating Scale (MADRS). FORWARD-5 achieved the primary endpoint and demonstrated that adjunctive BUP/SAM 2 mg/2 mg was superior to placebo (average difference change from baseline to week 3 through end of treatment [EOT] in MADRS-6 and −10 versus placebo: −1.5, *P* = 0.018*;* −1.9, *P* = 0.026, respectively). FORWARD-4 did not achieve the primary endpoint (change from baseline in MADRS-10 at week 5 versus placebo: –1.8, *P* = 0.109), although separate analyses showed significant treatment differences at other timepoints using traditional, regulatory-accepted endpoints such as reduction in MADRS-10 at EOT. The pooled analysis of the two studies demonstrated consistently greater reduction in MADRS-10 scores from baseline for BUP/SAM 2 mg/2 mg versus placebo at multiple timepoints including EOT and average change from baseline to week 3 through EOT (–1.8, *P* = 0.010; –1.8, *P* = 0.004, respectively). The overall effect size (Hedges’ *g*) in the pooled analyses for MADRS-10 change from baseline to EOT was 0.22. Overall, BUP/SAM was generally well tolerated, with most adverse events (AEs) being mild or moderate in severity. The most common AEs, occurring in ≥5% of patients in the BUP/SAM 2 mg/2 mg treatment group, which was more frequently than the placebo group, included nausea, constipation, dizziness, vomiting, somnolence, fatigue, and sedation. There was minimal evidence of abuse, and no evidence of dependence or opioid withdrawal by AEs or objective measures. This report describes adjunctive BUP/SAM 2 mg/2 mg combination, a therapy with a novel opioidergic mechanism of action, as a potential new treatment option for patients with MDD who have an inadequate response to currently available ADT.

## Introduction

Major depressive disorder (MDD) is associated with significant morbidity [1], and is a leading cause of global disability, affecting some 300 million people worldwide [2]. Predominant pharmacotherapies approved for treatment of MDD include selective serotonin reuptake inhibitors (SSRIs), selective serotonin and norepinephrine reuptake inhibitors (SNRIs), and bupropion [3], all of which target various aspects of monoaminergic neurotransmission, such as serotonin, norepinephrine, or dopamine transporters [4]. Many patients receiving these treatments do not respond adequately or fail to achieve remission; estimates suggest that only 37% of patients achieve remission after first-line therapy and that lower remission rates are observed for each subsequent step in treatment [4–8]. For these patients, treatments may include electroconvulsive therapy, repetitive transcranial magnetic stimulation, and cognitive therapy [8]. From a pharmacotherapy perspective, the only Food and Drug Administration-approved adjunctive therapies for these MDD patients are atypical antipsychotics, which also work through modulation of monoaminergic neurotransmission and can be associated with treatment-limiting adverse effects, including significant metabolic abnormalities and motor disorders, such as akathisia and the rare, but serious, event of tardive dyskinesia [4, 9]. Adverse events, particularly weight gain, may contribute to patients’ nonadherence to these medications. Since MDD is a complex syndrome that includes debilitating emotional, physical, and psychologic symptoms, impaired ability to cope with stress, and significant social dysfunction, which all contribute to diminished well-being [10–13], new agents with novel mechanisms of action are urgently needed [14].

The endogenous opioid system is a fundamental regulator of mood in humans and is thought to play a critical role in various functional and social processes affected by depression, including motivation, social functioning/attachment, and resiliency [15–18]. There is some evidence to support opioid modulation as a potential treatment target for MDD. Positron emission tomography imaging studies suggest opioidergic circuits are dysregulated in patients with MDD [19, 20]. Research also indicates that addressing endogenous opioid dysregulation in the setting of MDD may provide clinical benefits unique and distinct from existing depression treatments. Low-dose buprenorphine demonstrated antidepressant potential in open-label studies [21–24], and in a multicenter, double-blind, placebo-controlled study in patients with MDD [25]. However, the challenge of clinical use of opioids is their inherent risk for abuse and dependence [26].

Buprenorphine/samidorphan combination (BUP/SAM; ALKS 5461) is an investigational opioid system modulator for adjunctive treatment of MDD. BUP is a partial μ-opioid receptor agonist and κ-opioid receptor antagonist [27]. In-vivo, SAM has been demonstrated to function as a μ-opioid antagonist [28]. In-vitro, SAM binds with high affinity to human μ-, κ- and δ-opioid receptors and acts as an antagonist at μ-opioid receptors, with low-intrinsic activity at κ- and δ-opioid receptors [28]. The purpose of SAM in combination with BUP is to address the abuse and dependence potential of BUP, while preserving its antidepressant effects. Early-stage randomized, double-blind, placebo-controlled studies demonstrated antidepressant activity with BUP/SAM as adjunctive treatment in patients with MDD not responding adequately to SSRIs or SNRIs [14, 26].

The objective of the Focused On Results With A Rethinking of Depression (FORWARD)-4 and FORWARD-5 studies was to further evaluate the efficacy and safety of BUP/SAM as adjunctive treatment in MDD patients with inadequate response to continuing antidepressant therapy (ADT). Both studies utilized the sequential parallel-comparison design (SPCD) to mitigate the risk of excessive placebo responses common in MDD clinical trials [29, 30]. Efficacy and safety findings from the FORWARD-4 and FORWARD-5 studies, as well as pooled results, are reported here.

## Patients and methods

**Fig. 1:**
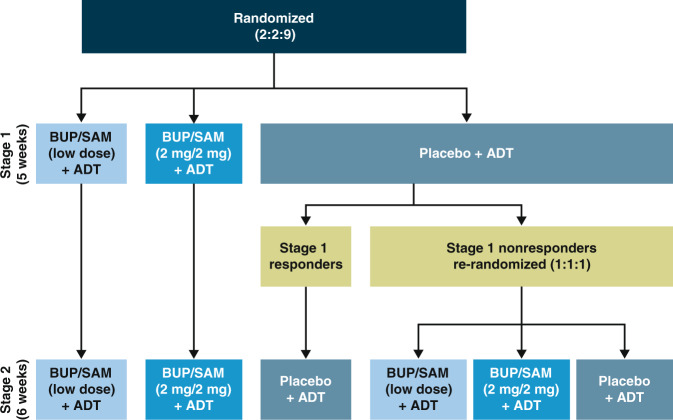
FORWARD-4 and FORWARD-5 study design. ADT antidepressant therapy; BUP buprenorphine; SAM samidorphan.

FORWARD-4 (ClinicalTrials.gov ID: NCT02158533) and FORWARD-5 (NCT02218008) were two global, phase III, multicenter, randomized, double-blind, placebo-controlled, SPCD studies conducted at 54 and 57 sites, respectively, evaluating BUP/SAM plus continued ADT. The studies were identical in design (Fig. 1), except for the timing and requirement of the safety follow-up visit. Both studies evaluated BUP/SAM 2 mg/2 mg dose. In addition, FORWARD-4 evaluated a 0.5 mg/0.5 mg dose and FORWARD-5 a 1 mg/1 mg dose. Treatment durations were 5 weeks for stage 1 and 6 weeks for stage 2. The same inclusion/exclusion criteria for eligibility, efficacy assessments, and frequency of treatment visits were utilized in both studies (see Supplementary Information for additional details). Both studies utilized enhanced blinding (masking) in which the overall study design, criteria for randomization, and points of randomization were blinded to the site investigators, study staff, and patients. Site investigators and study staff, including the statisticians, were blinded until database was locked for the studies. The sponsor designed the trial in collaboration with the authors and conducted the data analyses according to a statistical analysis plan (see Section Statistical analysis).

The study protocols were reviewed by an independent ethics committee or institutional review board at each site and conducted following the principles of Good Clinical Practice derived from the Declaration of Helsinki, and in accordance with local regulations and International Council of Harmonization guidelines.

### Patients

Female and male patients aged 18–70 years were eligible if they met *Diagnostic and Statistical Manual for Mental Disorders*, Fourth Edition, Text Revision criteria for MDD, their current major depressive episode (MDE) lasted 8 weeks to 24 months, and they experienced an inadequate response to one or two ADTs during the current MDE. Inadequate ADT response was defined as <50% reduction in symptom severity with an adequate antidepressant dose of an FDA-approved ADT for ≥8 weeks (including up to 3 weeks for titration into the adequate dose range and stable for ≥4 weeks), assessed by the Massachusetts General Hospital Antidepressant Treatment Response Questionnaire [31]. Inadequate responses were verified by remote raters reviewing historic records and/or prospectively collected response data. Additional study design details are described in the Supplementary information.

### Randomization and treatment stages

The hallmark of SPCD is the presence of two double-blind, placebo-controlled stages. In FORWARD-4 and FORWARD-5, patients entering stage 1 were randomized (2:2:9) to receive BUP/SAM 2 mg/2 mg, BUP/SAM low-dose (0.5 mg/0.5 mg or 1 mg/1 mg), or placebo administered as a once-daily sublingual tablet for 5 weeks with continued ADT (SSRI, SNRI, or bupropion) (Fig. 1). Patients assigned to BUP/SAM 2 mg/2 mg initiated treatment with the following 1-week blinded titration period: BUP/SAM 0.5 mg/0.5 mg for the first 3 days, BUP/SAM 1 mg/1 mg on days 4–7, and BUP/SAM 2 mg/2 mg thereafter. Patients assigned to BUP/SAM 1 mg/1 mg initiated treatment with BUP/SAM 0.5 mg/0.5 mg for the first 3 days and BUP/SAM 1 mg/1 mg thereafter. Patients assigned the 0.5 mg/0.5 mg dose did not undergo titration.

At the conclusion of stage 1, patients receiving placebo were blindly determined to be nonresponders if they had a Montgomery–Åsberg Depression Rating Scale (MADRS)-10 [32] score >15 at week 5 and a <50% reduction in MADRS-10 score from baseline to week 5. Placebo nonresponders were then rerandomized in stage 2 in 1:1:1 ratio to BUP/SAM 2 mg/2 mg, low-dose BUP/SAM, or placebo for a 6-week treatment period. Placebo responders from stage 1 remained on placebo in stage 2. Patients receiving BUP/SAM in stage 1 remained on the same dose during stage 2 (Fig. 1).

Patients could enter a long-term safety study of BUP/SAM (ClinicalTrials.gov ID: NCT02141399) immediately after the last treatment visit (FORWARD-5) or the safety follow-up visit (FORWARD-4). For both studies, treatment was stopped without dose tapering.

### Efficacy assessments

Efficacy assessments included two scores derived from the MADRS. MADRS-10 was the sum of all ten-items in the MADRS. MADRS-6 was the sum of six MADRS items representing core MDD symptoms as per Bech recommendations [33] (see Supplementary information). The MADRS was administered weekly. In both studies, longitudinal data were analyzed in statistical models to estimate change from baseline in MADRS score at each timepoint for the difference between BUP/SAM versus placebo within each stage. These estimates were averaged across the two stages using prespecified equal weights. Using these combined-stage estimates, the primary endpoint in FORWARD-4 was change from baseline to week 5 in MADRS-10. In FORWARD-5, three primary endpoints were defined hierarchically and tested sequentially to control Type 1 error for multiplicity as prespecified in the protocol and statistical analysis plan. Two of these endpoints analyzed the average difference between BUP/SAM and placebo in change from baseline to week 3 through end-of-treatment (EOT) using MADRS-6 and MADRS-10 scores. The third primary endpoint used change from baseline to EOT. To control for multiplicity due to multiple BUP/SAM doses, hypothesis tests were conducted in a prespecified order and a fixed sequence where the tests comparing BUP/SAM 2 mg/2 mg to placebo were conducted prior to those comparing lower BUP/SAM doses to placebo. These endpoints were also evaluated in FORWARD-4 as part of post hoc analysis.

Secondary efficacy endpoints for both studies included MADRS response (≥50% reduction in MADRS-10 score from baseline to week 5 in FORWARD-4 and EOT in FORWARD-5) and remission (MADRS-10 score ≤10 at week 5 and EOT, respectively).

An additional endpoint was the change in the clinician-rated Hamilton Anxiety Rating Scale (HAM-A) [34].

### Safety assessments

Safety and tolerability were assessed in all randomized patients who received at least one dose of study drug, based on adverse events (AEs), clinical laboratory parameters, and echocardiogram parameters. AEs of special interest (AESI) were assessed to evaluate abuse potential, dependence, and withdrawal, and AEs associated with suicidal ideation and/or behavior, sexual dysfunction, and hypomania/mania (see Supplementary information). Objective assessment of withdrawal used the clinical opiate withdrawal scale (COWS). Suicidal ideation and behavior was assessed at each visit with the Columbia-Suicide Severity Rating Scale (C-SSRS).

### Statistical analysis

Mixed models for repeated measures were used to assess change from baseline at each timepoint for all treatment arms as well as the BUP/SAM versus placebo difference during both stages of the SPCD study. As specified, some endpoints were based on single timepoint estimates and some on the average of estimates from multiple timepoints. Models included fixed effect variables for treatment group; visit; treatment group-by-visit interaction; site region; and site region-by-treatment interaction as categorical fixed effects, and baseline value and baseline-by-visit interaction as covariates. Random effects associated with patients were included as part of the marginal covariance matrix (specified as unstructured) as recommended for longitudinal data with continuous outcomes. Primary analyses were based on weighted combined-stage analysis using equal weights for BUP/SAM versus placebo difference derived from stage-specific models. Type 1 error due to multiplicity was controlled by testing each hypothesis (two-sided alpha = 0.05) in a prespecified fixed sequence for the primary endpoints. Efficacy analyses were performed using all randomized patients who received at least one dose of study drug and had at least one postbaseline MADRS measurement in the given stage. All statistical analyses on efficacy were conducted using SAS v9.4 (SAS Institute, Inc., Cary, NC). Sample size and power calculations were conducted using SAS v9.3 (SAS Institute, Inc., Cary, NC). A full description of the statistical analysis is provided in the Supplementary information.

In post hoc analyses, effect sizes (Hedges’ *g*) were calculated for each stage of the SPCD trial and for the stages combined as described in the Supplementary information.

The pooled analysis plan was prespecified following unblinding of FORWARD-4 and before unblinding of FORWARD-5, and utilized the same endpoints. The pooled efficacy analysis population included placebo and BUP/SAM 2 mg/2 mg treatment groups.

All safety assessments were summarized using descriptive statistics for each stage in the individual study and pooled safety populations.

## Results

### Patients

In FORWARD-4, 385 patients were randomized in stage 1 and 384 received at least one dose of study drug. All patients completing stage 1 (354; 92.2%) entered stage 2, of whom 168 were placebo nonresponders and rerandomized to placebo or BUP/SAM (Supplementary Figure 1A). Discontinuation rates during stage 1 were 13.3% in the BUP/SAM 2 mg/2 mg and 5.3% in the placebo group. During stage 2, discontinuation rates were 10.7% and 5.4%, respectively.

In FORWARD-5, 407 patients were randomized in stage 1 and 406 patients received at least one dose of study drug. Of 362 (89.2%) patients completing stage 1, 360 entered stage 2, including 187 placebo nonresponders who were rerandomized (Supplementary Figure 1b). Discontinuation rates during stage 1 were 23.8% in the BUP/SAM 2 mg/2 mg and 7.9% in the placebo group. During stage 2, discontinuation rates were 9.5% and 6.5%, respectively.

Demographic characteristics were generally similar between treatment groups in both studies (Table 1). Patients were predominantly Caucasian and female, with mean age ~45 years.

**Table 1:** Baseline demographic and clinical characteristics for randomized patients (safety population)

		FORWARD-4			FORWARD-5		
		Placebo + ADT *n* = 265	BUP/SAM (0.5 mg/0.5 mg) + ADT *n* = 59	BUP/SAM (2 mg /2 mg) + ADT *n* = 60	Placebo + ADT *n* = 280	BUP/SAM (1 mg /1 mg) + ADT *n* = 63	BUP/SAM (2 mg /2 mg) + ADT *n* = 63
*Patient demographics*							
Age (years), mean (s.d.)		45.8 (11.5)	45.0 (13.9)	46.2 (12.1)	45.7 (12.9)	45.1 (11.5)	42.9 (14.5)
Female, *n* (%)		182 (68.7)	38 (64.4)	40 (66.7)	193 (68.9)	42 (66.7)	42 (66.7)
Race, *n* (%)							
Caucasian		182 (68.7)	42 (71.2)	42 (70.0)	207 (73.9)	44 (69.8)	50 (79.4)
Black or African American		77 (29.1)	16 (27.1)	16 (26.7)	67 (23.9)	17 (27.0)	11 (17.5)
Other^a^		6 (2.3)	1 (1.7)	2 (3.3)	6 (2.1)	2 (3.2)	2 (3.2)
BMI (kg/m^2^), mean (s.d.)		30.3 (5.6)	30.1 (5.5)	29.8 (5.8)	29.2 (5.7)	29.9 (6.0)	28.7 (5.7)
*Characteristics of current MDE*							
MADRS total score, mean (s.d.)^b^		31.9 (5.0)	32.7 (4.7)	32.0 (5.7)	31.7 (5.6)	31.8 (5.3)	31.8 (5.6)
CGI-S score, mean (s.d.)^b^		4.6 (0.6)	4.5 (0.5)	4.6 (0.6)	4.6 (0.6)	4.6 (0.6)	4.7 (0.6)
HAM-D score, mean (s.d.)^b^		24.2 (3.3)	23.8 (3.6)	24.2 (3.8)	24.6 (3.7)	24.5 (3.4)	24.4 (3.5)
Duration of current MDE (months), mean (s.d.)		9.6 (5.8)	10.8 (6.0)	9.2 (5.0)	9.0 (5.5)	9.4 (5.2)	9.0 (5.3)
Classofantidepressant therapy, *n* (%)	SSRI	157 (59.2)	40 (67.8)	36 (60.0)	174 (62.1)	32 (50.8)	36 (57.1)
	SNRI	81 (30.6)	10 (16.9)	18 (30.0)	76 (27.1)	22 (34.9)	20 (31.7)
	Bupropion	27 (10.2)	9 (15.3)	6 (10.0)	30 (10.7)	9 (14.3)	7 (11.1)
*Disease history at randomization*							
No. of lifetime MDEs,^c^ *n* (%)	1	14 (5.3)	4 (6.8)	1 (1.7)	25 (8.9)	3 (4.8)	3 (4.8)
	2	49 (18.5)	9 (15.3)	11 (18.3)	48 (17.1)	13 (20.6)	14 (22.2)
	3–4	117 (44.2)	24 (40.7)	23 (38.3)	119 (42.5)	22 (34.9)	23 (36.5)
	>4	85 (32.1)	22 (37.3)	25 (41.7)	88 (31.4)	25 (39.7)	23 (36.5)
No. of lifetime ADTs, *n* (%)	1	47 (17.7)	15 (25.4)	9 (15.0)	61 (21.8)	13 (20.6)	10 (15.9)
	2	98 (37.0)	19 (32.2)	15 (25.0)	88 (31.4)	19 (30.2)	24 (38.1)
	>2	120 (45.3)	25 (42.4)	36 (60.0)	131 (46.8)	31 (49.2)	29 (46.0)

*ADT* antidepressant therapy; *BMI* body mass index; *BUP* buprenorphine; *CGI-S* Clinical Global Impression—Severity scale; *HAM-D* Hamilton Depression Rating Scale; *MDE* major depressive episode; *MADRS* Montgomery–Åsberg Depression Rating Scale; *SAM* samidorphan; *s.d.* standard deviation; *SNRI* serotonin and norepinephrine reuptake inhibitor; *SSRI* selective serotonin reuptake inhibitor

^a^Includes American Indian, Asian, Hawaiian, and Hispanic or Latino. ^b^*N* values differ from the safety population. ^c^Includes current episode

### Efficacy

**Fig. 2:**
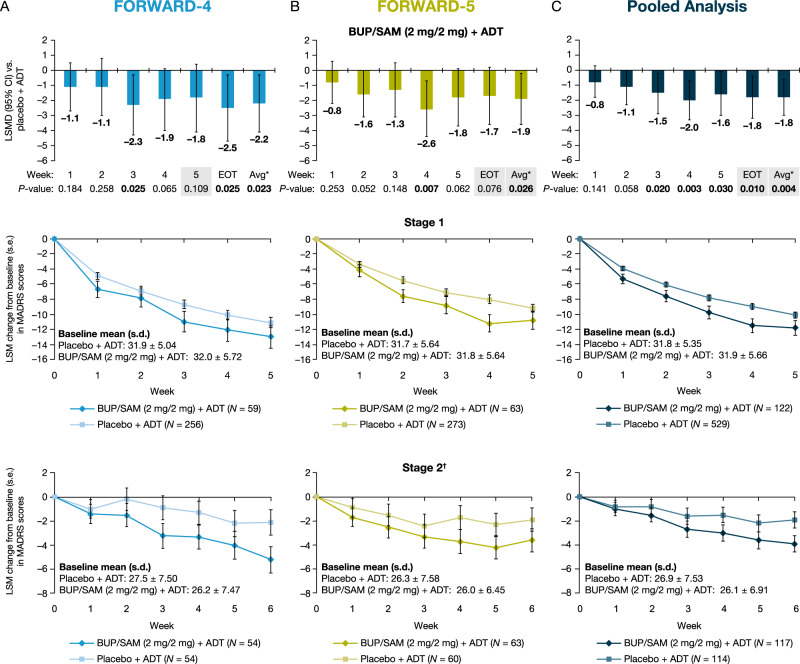
BUP/SAM (2 mg/2 mg) + ADT LSMD from placebo + ADT in the combined-stage change from baseline in MADRS-10 scores (first row) and LSM change in MADRS-10 scores for BUP/SAM (2 mg/2 mg) + ADT and placebo + ADT by week and stage (rows 2 and 3) in **A** FORWARD-4, **B** FORWARD-5, and **C** a pooled analysis. Shading indicates primary endpoints for each study. Error bars represent 95% CI or s.e. as indicated on the *y*-axis. *Avg: average change from baseline to week 3 through EOT. ^†^Change from stage 2 baseline. ADT antidepressant therapy; BUP buprenorphine; CI confidence interval; EOT end-of-treatment; LSM least squares mean; LSMD least squares mean difference; MADRS Montgomery–Åsberg Depression Rating Scale; SAM samidorphan; s.d. standard deviation; s.e. standard error

In FORWARD-4, the BUP/SAM 2 mg/2 mg group had a greater reduction in MADRS-10 score from baseline to week 5 than the placebo group; however, this primary endpoint was not statistically significant (least squares mean difference [LSMD]: –1.8; *P* = 0.109; 95% confidence interval [CI]: –4.1 to 0.4) (Fig. 2a). BUP/SAM 2 mg/2 mg did show numerically greater reduction in MADRS-10 scores than placebo at all timepoints in both stages (Fig. 2a). In post hoc analysis, BUP/SAM 2 mg/2 mg demonstrated greater reduction from baseline compared to placebo in MADRS-10 score to EOT (LSMD: –2.5; *P* = 0.025; 95% CI: –4.7 to –0.3) and average change from baseline to week 3 through EOT in MADRS-10 (LSMD: –2.2; *P* = 0.023; 95% CI: –4.1 to –0.3) (Fig. 2a) and MADRS-6 score (LSMD: –1.9; *P* = 0.004; 95% CI: –3.3 to –0.6) (Supplementary Figure 2A). The BUP/SAM 0.5 mg/0.5 mg group was not different from placebo at any timepoint, assessed by change in MADRS score (Supplementary Figure 3A). Changes in MADRS scores by stage and endpoint are shown in Table 2.

**Table 2:** Change in MADRS-6 and MADRS-10 scores from baseline by stage (LSMD versus placebo)

Endpoint	FORWARD-4				FORWARD-5				Pooled studies	
	Stage 1		Stage 2		Stage 1		Stage 2		Stage 1	Stage 2
	BUP/SAM (0.5 mg/0.5 mg) + ADT (*N* = 58)	BUP/SAM (2 mg/2 mg) + ADT (*N* = 59)	BUP/SAM (0.5 mg/0.5 mg) + ADT (*N* = 55)	BUP/SAM (2 mg/2 mg) + ADT (*N* = 54)	BUP/SAM (1 mg/1 mg) + ADT (*N* = 62)	BUP/SAM (2 mg/2 mg) + ADT (*N* = 63)	BUP/SAM (1 mg/1 mg) + ADT (*N* = 62)	BUP/SAM (2 mg/2 mg) + ADT (*N* = 63)	BUP/SAM (2 mg/2 mg) + ADT (*N* = 122)	BUP/SAM (2 mg/2 mg) + ADT (*N* = 117)
*MADRS-10 at baseline*										
Mean (s.d.)	32.7 (4.70)	32.0 (5.72)	26.6 (7.14)	26.2 (7.47)	31.8 (5.27)	31.8 (5.64)	27.7 (7.15)	26.0 (6.45)	31.9 (5.66)	26.1 (6.91)
*MADRS-10 change from baseline to EOT (stage 1: week 5 and stage 2: week 6)*										
LSMD versus placebo (s.e.)	2.7 (1.63)	–1.9 (1.65)	–2.4 (1.64)	–3.2 (1.53)	–1.1 (1.31)	–1.6 (1.34)	–1.5 (1.37)	–1.7 (1.37)	–1.7 (1.03)	–1.9 (0.98)
*P* value	0.097	0.255	0.151	0.041	0.382	0.220	0.281	0.203	0.099	0.048
*MADRS-10 average change from baseline score from week 3 through EOT*										
LSMD versus placebo (s.e.)	2.2 (1.45)	–2.1 (1.47)	–2.9 (1.40)	–2.3 (1.27)	–0.7 (1.15)	–2.2 (1.17)	–1.2 (1.26)	–1.6 (1.25)	–2.1 (0.91)	–1.5 (0.84)
*P* value	0.133	0.161	0.040	0.067	0.541	0.063	0.362	0.192	0.024	0.080
*MADRS-6 at baseline*										
Mean (s.d.)	21.9 (2.83)	20.8 (3.63)	17.9 (4.58)	17.8 (4.79)	21.2 (3.58)	21.3 (4.06)	18.5 (4.66)	17.9 (4.66)	21.1 (3.86)	17.8 (4.70)
*MADRS-6 average change from baseline score from week 3 through EOT (stage 1: week 5 and stage 2: week 6)*										
LMD versus placebo (s.e.)	1.5 (1.02)	–1.4 (1.04)	–2.2 (0.96)	–2.5 (0.87)	–0.5 (0.81)	–1.3 (0.83)	–0.7 (0.93)	–1.7 (0.93)	–1.4 (0.65)	–1.6 (0.60)
*P* value	0.153	0.170	0.021	0.005	0.565	0.123	0.430	0.075	0.034	0.008
*MADRS-6 change from baseline to EOT (stage 1: week 5 and stage 2: week 6)*										
LSMD versus placebo (s.e.)	1.9 (1.16)	–1.3 (1.17)	–1.9 (1.16)	–3.3 (1.08)	–0.6 (0.95)	–0.8 (0.97)	–1.0 (1.02)	–1.9 (1.01)	–1.1 (0.74)	–2.1 (0.72)
*P* value	0.102	0.261	0.095	0.003	0.551	0.426	0.331	0.068	0.150	0.004

*ADT* antidepressant therapy; *BUP* buprenorphine; *EOT* end-of-treatment; *LSMD* least squares mean deviation; *MADRS* Montgomery–Åsberg Depression Rating Scale; *SAM* samidorphan; *s.d.* standard deviation; *s.e.* standard error

For FORWARD-4, a post hoc analysis included the change in MADRS-10 score from baseline to EOT, and the average change from baseline score from week 3 through EOT, and the change in MADRS-6 score from baseline to week 3 to EOT. MADRS-10 is the sum of all 10 items of the scale and ranges from 0 to 60. The 10 items are: reported sadness, apparent sadness, inner tension, reduced sleep, reduced appetite, concentration difficulties, lassitude, inability to feel, pessimistic thoughts, and suicidal thoughts. MADRS-6 is the sum of the following six items: reported sadness, apparent sadness, inner tension, lassitude, inability to feel, and pessimistic thoughts

In FORWARD-5, the BUP/SAM 2 mg/2 mg group met the primary endpoints of the average change from baseline to week 3 through EOT for MADRS-6 (LSMD: –1.5; *P* = 0.018; 95% CI: –2.7 to –0.3) (Supplementary Figure 2B) and MADRS-10 (LSMD: –1.9; *P* = 0.026; 95% CI: –3.6 to –0.2) (Fig. 2b). The change from baseline to EOT in MADRS-10 numerically favored BUP/SAM 2 mg/2 mg versus placebo, but did not reach statistical significance (LSMD: –1.7; *P* = 0.076; 95% CI: –3.6 to 0.2) (Fig. 2b). BUP/SAM 2 mg/2 mg had a numerically greater reduction than placebo in MADRS-10 scores at all timepoints during both stages (Fig. 2b). Unlike the BUP/SAM 0.5 mg/0.5 mg group in FORWARD-4, the BUP/SAM 1 mg/1 mg group in FORWARD-5 showed numerically greater improvement compared with placebo in MADRS scores, but did not reach statistical significance (Supplementary Figure 3B). Changes in MADRS scores by stage and endpoint are shown in Table 2.

In both studies, the secondary efficacy endpoints of rates of MADRS treatment response and remission were numerically higher in the BUP/SAM 2 mg/2 mg than placebo group; however, differences were not statistically significant (Supplementary Table 1). No statistically significant differences in HAM-A scores were observed in the BUP/SAM dose groups relative to placebo with treatment.

In the pooled analysis, change from baseline on the MADRS-10 in the BUP/SAM 2 mg/2 mg group was greater compared with placebo at all timepoints from week 3 and later, including EOT (LSMD: –1.8; *P* = 0.010; 95% CI: –3.2 to –0.4), as well as the average change from baseline to week 3 through EOT (LSMD: –1.8; *P* = 0.004; 95% CI: –3.0 to –0.6) (Fig. 2c). Changes from baseline in the pooled analysis are shown by stage in Fig. 2c. MADRS-6 scores were also reduced compared to placebo at EOT during stage 1 and 2 (Table 2). Effect sizes (Hedges’ *g*) in the pooled analyses for MADRS-10 change from baseline to EOT increased from 0.17 in stage 1 to 0.26 in stage 2, with an overall pooled value of 0.22; the overall value was 0.23 for MADRS-10 average change from baseline to week 3 through EOT, similar to the individual studies (Supplementary Table 2). The effect size was greater for MADRS-6 average change from baseline to week 3 through EOT (0.29). Additional information on effects up to 11 weeks is available in the Supplementary Information (Supplementary Figure 4).

### Safety

The safety profile for BUP/SAM was similar for the studies and no dose-dependent effects were observed (Supplementary Table 3). In the pooled study group, overall incidences of treatment-emergent AEs during stage 1 were 67.5% in the BUP/SAM 2 mg/2 mg and 53.8% in the placebo group. Incidences of serious AEs were low in both groups (BUP/SAM 2 mg/2 mg: 1.6%; placebo: 0.4%). In stage 2, the overall incidence of AEs was lower than in stage 1, with corresponding rates of 45.4 and 45.8% for BUP/SAM 2 mg/2 mg and placebo, respectively (Table 3). Most AEs were mild or moderate in severity in both stages, occurred at treatment initiation, and resolved with continued treatment. Among patients treated with BUP/SAM 2 mg/2 mg, 14.6% and 2.5% of AEs led to study discontinuation in stage 1 and stage 2, respectively. AEs occurring in ≥5% of patients in the BUP/SAM 2 mg/2 mg group and more frequently than the placebo group included nausea, constipation, dizziness, vomiting, somnolence, fatigue, and sedation in stage 1. The only AE occurring in ≥5% of patients in the pooled BUP/SAM 2 mg/2 mg group and more frequently than placebo in stage 2 was nausea. There were no clinically meaningful changes in vital signs across groups, including respiratory rate, pulse, and blood pressure, as well as body weight. Changes in laboratory and echocardiogram parameters were small and not clinically meaningful.

**Table 3:** Safety events among patients in the pooled analysis of FORWARD-4 and FORWARD-5

Event, *n* (%)	Stage 1		Stage 2	
	Placebo + ADT (*n* = 545)	BUP/SAM (2 mg/2 mg) + ADT (*n* = 123)	Placebo + ADT (*n* = 118)	BUP/SAM (2 mg/2 mg) + ADT (*n* = 119)
Any AE	293 (53.8)	83 (67.5)	54 (45.8)	54 (45.4)
Any SAE	2 (0.4)	2 (1.6)	1 (0.8)	0
AE leading to study discontinuation	12 (2.2)	18 (14.6)	2 (1.7)	3 (2.5)
*Common AEs*^a^				
Nausea	37 (6.8)	34 (27.6)	2 (1.7)	13 (10.9)
Constipation	13 (2.4)	15 (12.2)	1 (0.8)	6 (5.0)
Dizziness	21 (3.9)	15 (12.2)	2 (1.7)	4 (3.4)
Vomiting	11 (2.0)	12 (9.8)	1 (0.8)	5 (4.2)
Headache	44 (8.1)	10 (8.1)	5 (4.2)	4 (3.4)
Somnolence	19 (3.5)	9 (7.3)	1 (0.8)	0
Fatigue	6 (1.1)	9 (7.3)	2 (1.7)	4 (3.4)
Sedation	4 (0.7)	8 (6.5)	0	0
Any AESI of abuse potential	43 (7.9)	30 (24.4)	3 (2.5)	4 (3.4)
*Euphoria related*				
Feeling abnormal	1 (0.2)	2 (1.6)	0	0
Euphoric mood	0	1 (0.8)	0	0
Feeling of relaxation	0	1 (0.8)	0	0
*Nonspecific*				
Dizziness	21 (3.9)	15 (12.2)	2 (1.7)	4 (3.4)
Somnolence	19 (3.5)	9 (7.3)	1 (0.8)	0
Sedation	4 (0.7)	8 (6.5)	0	0
Disturbance in attention	1 (0.2)	0	0	0

*ADT* antidepressant therapy; *AE* adverse event; *BUP* buprenorphine; *SAE* serious adverse events; *SAM* samidorphan

^a^Occurring in ≥5% of patients in the BUP/SAM 2 mg/2 mg treatment groups

The incidence of AESIs to evaluate abuse potential was low in both treatment groups in the pooled analysis, with similar outcomes in the individual studies. In stage 1, four (3.2%) patients who received BUP/SAM 2 mg/2 mg and one (0.2%) who received placebo reported AEs possibly euphoria related (Table 3; Supplementary Table 3). The majority of these AESIs were nonspecific events including sedation, reported by 6.5% of patients receiving BUP/SAM 2 mg/2 mg and 0.7% receiving placebo, somnolence in 7.3% and 3.5%, respectively, and dizziness in 12.2% and 3.9%, respectively. No patient who received BUP/SAM reported an AE associated with abuse behavior or dependence. There was no evidence of opioid withdrawal assessed by AESIs and COWS. Incidences of AEs to evaluate opioid withdrawal were similar following study drug discontinuation in both groups (Supplementary Table 4). Changes from final treatment visit to postdiscontinuation visit on mean COWS scores were low and similar between treatment groups (mean [s.d.] 0.3 [2.0] BUP/SAM 2 mg/2 mg and 0.1 [1.4] placebo) (Supplementary Table 5).

There were no completed suicides nor incidents of suicidal behavior or serious suicidal ideation in patients receiving BUP/SAM 2 mg/2 mg. Based on C-SSRS results, the incidence of suicidal ideation was lower in patients receiving BUP/SAM 2 mg/2 mg than placebo in both stage 1 and 2 (Supplementary Table 6). There was no evidence of hypomania/mania based on review of AESIs.

## Discussion

The data from these two FORWARD trials support the view that the BUP/SAM combination represents a promising potential adjunctive treatment for patients with MDD, acting through a novel, opioidergic mechanism of action compared with current antidepressant therapies. In the FORWARD-5 study, adjunctive BUP/SAM 2 mg/2 mg consistently reduced depression symptomatology compared to placebo across multiple timepoints in patients continuing their current ADT and met the primary endpoints of reducing core and overall depression symptoms. In FORWARD-4, the primary endpoint of change in MADRS-10 from baseline to week 5 was not statistically significant; however, reductions in symptom scores at multiple timepoints are consistent with the observed efficacy in FORWARD-5. Pooled analysis of the BUP/SAM 2 mg/2 mg arms, which provides a more precise estimate of treatment effect using a larger dataset, consistently demonstrated efficacy of the BUP/SAM 2 mg/2 mg arms versus placebo. In addition, the treatment effects observed at the various doses of BUP/SAM indicated a dose–response relationship.

Increasing placebo response rates in outpatient depression studies observed over the past several decades have made it more difficult to detect antidepressant efficacy and determine effect size [35, 36]. For example, several recently completed phase III studies of new adjunctive agents for MDD, including agents with known efficacy, have failed to meet primary endpoints [37–43]. While some studies were considered supportive of a therapeutic effect [38, 40], the majority found no evidence of efficacy [37, 39, 42, 43], highlighting the challenge encountered in clinical trials, especially outpatient studies, for new antidepressant treatments. To minimize the risk of excessive placebo response rates, the FORWARD-4 and FORWARD-5 studies were conducted utilizing SPCD as a strategy to increase statistical power in smaller sample sizes [44, 45] and enhance signal detection [14, 45, 46]. Consistent with the intent of using SPCD, the placebo response rate for the change from baseline in MADRS-10 score was reduced by ~40% from stage 1 (26.5%) to stage 2 (10.5%) in the pooled analyses. Nevertheless, it is possible that smaller sample sizes utilized in the SPCD design result in greater week-to-week variability in assessments due to fluctuations in symptomatology commonly observed in patients with MDD. Such variability may have been a factor in the inability to observe a statistically significant difference between BUP/SAM and placebo at the prespecified single timepoint (week 5) in FORWARD-4. This observation informed the prespecified analysis plan for FORWARD-5, where approaches such as averaging multiple weeks were employed to reduce the impact of week-to-week variability and allowed greater precision in determination of a treatment effect. This type of analysis is customary in trials of therapies for other conditions, such as pain, where typically the average of several datapoints is used as the endpoint [47].

The effect size (Hedges’ *g*) for MADRS-10 (change from baseline to EOT) was greater in stage 2 (0.26) compared with stage 1 (0.17), indicating a greater ability to detect a treatment effect and consistent with a lower placebo effect in stage 2. Additionally, the effect sizes observed with BUP/SAM 2 mg/2 mg are consistent with those observed with antidepressants, including adjunctive therapies in MDD [48].

Taken together, these findings indicate evidence of an antidepressant effect for BUP/SAM 2 mg/2 mg and support utilization of study design methodologies such as SPCD to mitigate risk of excessive placebo response rates in psychiatric studies.

It is noteworthy that the ten-item MADRS scale was developed to detect changes with tricyclic antidepressants available in the 1970s [32], and has subsequently been utilized in a large number of monoamine-based drug development programs [48]. Given the unique mechanism of BUP/SAM, investigating treatment effects on domains specifically related to the endogenous opioid system—such as resiliency, motivation, and social attachment—may achieve a more comprehensive understanding of the therapeutic effects of this therapy than using MADRS alone, but these were not in the scope of the FORWARD studies.

MADRS response and remission rates in both FORWARD studies were numerically greater in the BUP/SAM 2 mg/2 mg group, although not statistically significantly different from placebo. The duration of each SPCD stage may have been insufficient to show separation between groups in response or remission and may require assessments over longer periods. Compared to the BUP/SAM phase II study [14], MADRS response and remission rates were lower in both FORWARD studies. A similar phenomenon was observed in the recent Delphinus study where, following 6 weeks of therapy, response and remission rates were 10.5% and 6.8%, respectively, for adjunctive brexpiprazole and 8.1% and 2.0% for adjunctive quetiapine, compared with 6.8% and 4.4% for ADT plus placebo [38]. Like the FORWARD studies, Delphinus identified nonresponders to ADT prior to randomization to active adjunctive treatment and incorporated measures of masking to ensure investigators were unaware of the timing of treatment initiation. The authors hypothesized that additional masking measures may explain, in part, the lower response and remission rates observed in the randomized treatment phase than previous studies of brexpiprazole. The data from these clinical trials inform treatment of MDD; however, as MDD is a chronic disorder, data from long-term trials will be important to evaluate efficacy and safety over months of treatment. Treatment length is a limitation of these two studies.

BUP/SAM 2 mg/2 mg was generally well tolerated in these studies, with most AEs mild/moderate in severity and not leading to treatment discontinuation. As an adjunctive therapy for MDD, BUP/SAM was not associated with metabolic disturbances, motor disorders, evidence of induction of hypomania/mania, or sexual dysfunction. There was also no evidence of increased risk for suicidal ideation or behavior observed with BUP/SAM 2 mg/2 mg, based on AEs or C-SSRS scores. Patients at risk of imminent suicide were excluded. However, these studies allowed patients with suicidal ideation without intent. Hence, these data would be informative in the treatment of outpatients with MDD.

In the pooled analysis, there was a lower incidence of suicidal ideation with adjunctive BUP/SAM compared with ADT alone—an intriguing finding that warrants further exploration considering its potential importance to patients with MDD.

BUP, along with other μ-opioid receptor agonists, has established abuse liability [49], and SAM was included in the combination to address this issue [26]. The incidence of euphoric events with BUP/SAM was low within these two studies and there were no reports of abuse behavior. In addition, there was no evidence of dependence or opioid withdrawal by AEs or objective measures, indicating that SAM is acting as intended to mitigate the risk of abuse and dependence associated with BUP.

These results provide consistent evidence of the efficacy of adjunctive BUP/SAM in patients with MDD inadequately responding to antidepressants. BUP/SAM 2 mg/2 mg was generally well tolerated and showed low potential for abuse. As currently available antidepressant and adjunctive therapies generally target monoamine-based neural signaling, for the substantial population of patients with MDD who have not achieved adequate symptom control with these therapies, a new class of antidepressant with a novel mechanism of action, such as opioid system modulation with BUP/SAM, could have significant impact.

## Electronic supplementary material

Supplementary Information

Supplementary Figure 1A

Supplementary Figure 1B

Supplementary Figure 2

Supplementary Figure 3

Supplementary Figure 4
