# Supplementary material for: Opioid system modulation with buprenorphine/samidorphan combination for major depressive disorder: two randomized controlled studies
Source: Mol Psychiatry. 2018 Oct 29;25(7):1580–91. doi: 10.1038/s41380-018-0284-1 (PMC7303008; doi:10.1038/s41380-018-0284-1)
Supplement: Supplementary file 1 — Supplementary Information [file 41380_2018_284_MOESM1_ESM.docx]

**SUPPLEMENTARY INFORMATION**

## Summaries

***Word documents***

**Supplementary Information:** Methods.

Summary: The Supplementary Information: Methods file provides further information on the methods used for the two studies, including the verification of inadequate response, inclusion and exclusion criteria, MADRS-10 and MADRS-6 definitions, safety assessments, and statistical analysis.

**Supplementary Table 1.** MADRS-10 treatment response and remission rates at EOT (LOCF) in FORWARD-4, FORWARD-5, and the pooled analysis.

Summary: Supplementary Table 1 presents the treatment response and remission rates at EOT for the FORWARD-4, FORWARD-5, and the pooled analysis.

**Supplementary Table 2.** Summary of effect size of BUP/SAM (2 mg/2 mg) + ADT in FORWARD-4, FORWARD-5, and the pooled analysis.

Summary: Supplementary Table 2 presents the effect size of BUP/SAM (2 mg/2 mg) + ADT in the FORWARD-4, FORWARD-5, and the pooled analysis for the average change in baseline from week 3 to EOT for MADRS-6 and MADRS-10 and the change from baseline at EOT for MADRS-10.

**Supplementary Table 3.** Safety events among patients in FORWARD-4 and FORWARD-5.

Summary: Supplementary Table 3 presents the safety events, occurring in ≥ 2% of patients in any of the BUP/SAM treatment groups, among patients in FORWARD-4 and FORWARD-5.

**Supplementary Table 4.** Postdiscontinuation emergent AEs associated with withdrawal in the pooled analysis.

Summary: Supplementary Table 4 presents any postdiscontinuation event associated with withdrawal in the pooled analysis.

**Supplementary Table 5.** Summary of COWS scores in FORWARD-4, FORWARD-5, and the pooled analysis.

Summary: Supplementary Table 5 presents the COWS scores at early termination, the change in COWS score from early termination to follow-up visit, and the COWS score by category at early termination.

**Supplementary Table 6.** Summary of suicidal behavior and ideation during stage 1 and stage 2 in FORWARD-4 and FORWARD-5 (C-SSRS).

Summary: Supplementary Table 6 presents the rates of suicidal behavior and ideation during stage 1 and stage 2 in FORWARD-4 and FORWARD-5.

**Figure files**

**Supplementary Figure 1.** Patient disposition in (A) FORWARD-4 and (B) FORWARD-5.

Summary: Supplementary Figure 1A presents the patient disposition for the FORWARD-4 study and Supplementary Figure 1B presents the patients disposition for the FORWARD-5 study.

**Supplementary Figure 2.** BUP/SAM (2 mg/2 mg) + ADT LSMD from placebo + ADT in the change from baseline in MADRS-6 scores across the entire study in (A) FORWARD-4, (B) FORWARD-5, and (C) the pooled analysis. Shading indicates primary endpoints for each study.

Summary: Supplementary Figure 2 presents the BUP/SAM (2 mg/2 mg) + LSMD from placebo + ADT in the change from baseline in MADRS-6 scores across the entire study.

**Supplementary Figure 3.** LSMD from placebo in the change from baseline in MADRS scores across the entire study for (A) BUP/SAM (0.5 mg/0.5 mg) + ADT in FORWARD-4 and (B) BUP/SAM (1 mg/1 mg) + ADT in FORWARD-5. Shading indicates primary endpoints for each study.

Summary: Supplementary Figure 3 presents the low dose BUP/SAM (0.5 mg/0.5 mg or 1 mg/1 mg) + ADT LSMD from placebo + ADT in the change from baseline in MADRS-10 and MADRS-6 scores across the entire study.

**Supplementary Figure 4.** Mean change from baseline MADRS-10 scores by visit during the entire treatment period for the BUP/SAM (2 mg/2 mg) + ADT and placebo + ADT treatment groups in (A) FORWARD-4 and (B) FORWARD-5 stage 1 full analysis sets.

Summary: Supplementary Figure 4 presents the efficacy of BUP/SAM (2 mg/2 mg) + ADT compared with placebo + ADT in the stage 1 full analysis set over the entire 11-week studies.

# SUPPLEMENTARY INFORMATION: METHODS

## Verification of inadequate response

Inadequate responses to prior therapy were verified by historic records or by prospectively collected response data. Patients were termed historical inadequate responders if their medical records indicated the following criteria:

1. An inadequate response (i.e., nonresponse) to their current antidepressant therapy (ADT)
2. No more than two inadequate responses to an ADT in the current major depressive episode (MDE; including the current inadequate response)
3. A 17-item Hamilton Rating Scale for Depression (HAM-D) total score of ≥18.

Patients not currently on ADT could participate in an 8-week prospective lead-in (PLI) period, during which they received open-label treatment with one of the following: escitalopram, sertraline, duloxetine, venlafaxine, bupropion, or fluoxetine, at the discretion of the Investigator and had no more than one previous inadequate response to ADT in the current MDE. At the end of the PLI period, patients designated as prospective inadequate responders and who had met all entry criteria continued to randomization.

## Inclusion and exclusion criteria

Patients meeting the following criteria were considered for admission to the study:

1. Willing and able to provide informed consent
2. Aged between 18 and 70 years
3. Had a body mass index of 18.0–40.0 kg/m^2^
4. Agreed to use an acceptable method of contraception for the duration of the study, unless surgically sterile or postmenopausal
5. Had a *Diagnostic and Statistical Manual for Mental Disorders*, Fourth Edition, Text Revision (DSM-IV-TR) major depressive disorder (MDD) primary diagnosis as assessed and confirmed by the Mini International Neuropsychiatric Interview (MINI; administered by qualified site staff). The primary diagnosis was defined as the primary source of current distress and functional impairment, in the opinion of the Investigator
6. Had a current MDE lasting 8 weeks to 24 months
7. Willing and able to follow the study procedures as outlined in the protocol, including adherence with both the approved ADT and the study drug regimen.

In addition, for those patients entering the PLI period, the following criteria were considered for admission to the study:

1. Not currently taking any ADT
2. Had no more than one inadequate response (<50% reduction in depressive symptom severity during a course of treatment at an adequate dose ≥8 weeks’ duration with the same, adequate dose of ADT in the last 4 weeks, as assessed by the Massachusetts General Hospital Antidepressant Treatment Response Questionnaire [ATRQ]) to ADT in the current MDE, as of screening
3. Had a HAM-D total score ≥22 at screening.

For those patients bypassing the PLI period (historic inadequate responders), the following criteria were considered for admission to the study:

1. Had been treated with an adequate dose of a selective serotonin reuptake inhibitor (SSRI), selective serotonin and norepinephrine reuptake inhibitor (SNRI), or bupropion during the current MDE for ≥8 weeks, with the same, adequate dose over the last 4 weeks that was expected to remain stable throughout the study
2. Had no more than two inadequate responses to ADT (inclusive of current inadequate response) in the current MDE, as of screening. Current inadequate response must have been to a SSRI, SNRI, or bupropion. Prior inadequate response within the current MDE, if applicable, may have been to any commercially available ADT
3. Had an inadequate response (<50% reduction in depressive symptom severity during a course of treatment ≥8 weeks’ duration with an adequate dose of a SSRI, SNRI, or bupropion, as assessed by the ATRQ) to current ADT treatment
4. Had a HAM-D total score ≥18 at screening.

Patients were deemed eligible for randomization by independent qualification, as evidenced by an independent-rater administered SAFER (state versus trait, assessability, face validity, ecologic validity, and the ‘rule of three Ps’ [pervasive, persistent, and pathologic]) interview, ATRQ, and a HAM-D total score ≥18 and Clinical Global Impression—Severity Scale score ≥4 at baseline. Patients who had participated in the PLI period were considered eligible for randomization if they had been treated during the PLI period with an adequate dose of escitalopram, sertraline, duloxetine, venlafaxine, bupropion, or fluoxetine for ≥8 weeks, with the same adequate dose over the last 4 weeks, and had met additional masked criteria for prospective inadequate response as determined by an interactive voice-/web-response system.

Patients were excluded from the study if they met any of the following criteria:

1. Had any finding that in the view of the Investigator would compromise the safety of the patient or affect his/her ability to adhere to the protocol visit schedule or fulfill visit requirements
2. Had any current primary Axis I diagnosis other than MDD, where primary diagnosis was defined as the primary source of current distress and functional impairment, in the opinion of the Investigator
3. Had any of the following psychiatric conditions per DSM-IV-TR criteria, as assessed by the MINI: lifetime history of an Axis I diagnosis of delirium, dementia, schizophrenia, or other psychotic disorder (including psychotic depression), or bipolar I or II disorder; history within the past 12 months of an Axis I diagnosis of eating disorder, obsessive-compulsive disorder, panic disorder, posttraumatic stress disorder, or acute stress disorder; clinically significant current Axis II diagnosis of borderline, antisocial, paranoid, schizoid, schizotypal, or histrionic personality disorder; or current diagnosis or clinical evidence of any cognitive disorder at screening
4. Had experienced hallucinations, delusions, or any psychotic symptoms in the current MDE
5. Had been hospitalized with a condition related to MDD within 3 months before screening
6. Had initiated psychotherapy within 6 weeks of screening or had an anticipated need for initiating psychotherapy during the study. A stable course of psychotherapy initiated >6 weeks prior to screening was permitted to continue throughout the study
7. Had used any of the following: an antipsychotic (e.g., aripiprazole, quetiapine, olanzapine) at any dose or duration for any indication within 1 year of screening or within the current MDE (whichever is longer); a nonantipsychotic adjunctive treatment (e.g., lithium, bupropion, psychostimulants) for the purpose of augmenting the effects of an ADT at any dose for a duration ≥6 weeks during the current MDE; any adjunctive treatment for the purpose of augmenting the effects of an ADT at any dose or duration within 30 days of screening; bupropion for smoking cessation at any dose for a duration of ≥6 weeks during the current MDE, or for any duration within 30 days of screening; an anticonvulsant (e.g., topiramate, gabapentin, lamotrigine, oxcarbazepine) at any dose or duration for any indication within 1 year of screening; opioid agonists (e.g., codeine, oxycodone, tramadol, morphine) or opioid antagonists (e.g., naloxone, naltrexone) within 14 days prior to screening, had anticipated a need for opioid use at any point during the study (e.g., planned surgery), or had used an extended-release formulation of an opioid antagonist within 2 months prior to screening
8. Had used any of the following: a course of pharmacotherapy (including prescription or over-the-counter medications) or natural supplements for insomnia, if initiated within 30 days of screening. Prescription, over-the-counter medications, and natural supplements were permitted to treat insomnia if they had been used stably for ≥30 days prior to screening, not more than three times per week, and were expected to be used stably at no more than three times per week throughout the study; a hypnotic agent for any psychiatric indication other than insomnia within 30 days of screening; any pharmacotherapy (including prescription or over-the-counter medications) or natural supplements for anxiety within 30 days of screening
9. Had initiated or had a dose adjustment to hormone replacement therapy (including testosterone) or oral contraceptive within 30 days of screening
10. Had used inducers or moderate to strong inhibitors of cytochrome P450 (CYP) 3A4 (prescription medications, over-the-counter medications, or dietary supplements) within 30 days prior to screening
11. Had received electroconvulsive therapy treatment within the last 5 years or received more than one course of electroconvulsive treatment during the patient’s lifetime
12. Posed a current suicide risk as evidenced by any of the following: it was the opinion of the investigator that the patient may have been at risk for suicide; the patient responded ‘Yes’ to the baseline Columbia-Suicide Severity Rating Scale (C-SSRS) Question 4 (‘Active Suicidal Ideation with Some Intent to Act, Without Specific Plan’) or Question 5 (‘Active Suicidal Ideation with Specific Plan and Intent’) if the most recent episode occurred within the past 12 months; the patient had attempted suicide within the past 2 years
13. Had a QT interval >450 msec for males and >470 msec for females, assessed in a relaxed state, as corrected by the Fridericia formula (QTcF) observed at visit 1 or 2
14. Had an aspartate aminotransferase or alanine transaminase measurement of >2 × the upper limit of normal at visit 1
15. Had current evidence of or history of any of the following: compromised respiratory function (e.g., chronic obstructive pulmonary disease, respiratory depression, signs or symptoms of hypoxia at screening); thyroid pathology (unless stabilized and euthyroid for >3 months at the time of screening); seizure disorder (excluding febrile seizure); hepatitis B, hepatitis C, or human immunodeficiency virus infection; myasthenia gravis; or any contraindicated medical condition as per the approved labeling for buprenorphine (BUP)
16. Had current evidence of or a history (in the past 12 months) of alcohol or substance abuse or dependence (excluding nicotine) per DSM-IV-TR criteria, as assessed by the MINI
17. Had a positive breath alcohol test at screening
18. Had a positive test for drugs of abuse at screening or visit 2 (exception: a positive screen for benzodiazepine may not have been exclusionary if such medication was medically indicated for insomnia)
19. For female patients, was pregnant, planning to become pregnant, or breastfeeding during the study
20. Had a history of either of the following: intolerance, allergy, or hypersensitivity to opioid antagonists (e.g., BUP, oxycodone) or opioid antagonists (e.g., naltrexone, naloxone); or nausea and/or vomiting when taking an opioid agonist or opioid antagonist that interfered with the ability to continue on study drug
21. Had a significant blood loss (>500 ml) or blood donation (including platelets or plasma) within 60 days of screening or between screening and randomization or had anticipated blood donation at any time during the study
22. Had participated in any of the following: clinical studies of more than two distinctive investigational products with a central nervous system indication in the past 4 years; any clinical study of an investigational product given as an adjunctive treatment for MDD at any time during the current MDE; and/or any clinical study of an investigational product and/or received an investigational drug or device within 30 days of screening
23. Had participated in a prior clinical study of BUP/samidorphan (SAM)
24. Was an employee of the investigator or study center, or immediate family of such employees or the investigator
25. Was an employee or immediate family of an employee (permanent, temporary contract worker, or designee responsible for the conduct of the study) of Alkermes or INC Research.

## MADRS-10 and MADRS-6 definition

The Montgomery–Åsberg Depression Rating Scale (MADRS)-10 was defined as the sum of responses to the following 10 items: apparent sadness, reported sadness, inner tension, reduced sleep, reduced appetite, concentration difficulties, lassitude, inability to feel, pessimistic thoughts, and suicidal thoughts.

MADRS-6 was defined as the sum of the six core symptoms domains (reported sadness, apparent sadness, inner tension, lassitude, inability to feel, and pessimistic thoughts) of the MADRS instrument.

## Method of assigning patients to treatment

Beginning with Visit 2 (stage 1 baseline), randomization and treatment assignment was done via an independent interactive voice or web response system (IxRS). Once a randomization number was assigned, that number was not used again. Codes were prepared by an independent biostatistician who was not otherwise involved in this study. Randomization, eligibility, and treatment assignment at Visit 7 (stage 2 baseline) was determined via an independent IxRS.

## Safety assessments

An adverse event (AE) was considered treatment-emergent if it started or worsened (if present at baseline) on or after the baseline during the relevant safety period). AEs were coded by preferred terms and system organ class using the Medical Dictionary for Regulatory Activities version 18.0.

AEs of special interest to evaluate abuse potential included euphoric mood, mood altered, elevated mood, inappropriate affect, hallucination, auditory hallucination, visual hallucination, feeling drunk, feeling abnormal, and sedation. AEs associated with suicidal ideation and/or behavior included completed suicide, depression suicidal, intentional overdose, intentional self-injury, poisoning deliberate, self-injurious behavior, self-injurious ideation, suicidal behavior, suicidal ideation, and suicide attempt.

## Statistical analysis

For both studies, a sample size of ~350 patients was estimated to provide ~90% power to detect a treatment difference between active and placebo MADRS change from baseline. Sample size and power were determined by using simulated longitudinal data and SAS v9.3 (SAS Institute, Inc., Cary, NC). Analyses were repeated on 5000 simulated datasets under a range of assumptions for MADRS change from baseline, correlation in repeated longitudinal data, timing and rate of discontinuation, and sample size. Power was calculated as the percent of simulated datasets that rejected the null hypothesis of no difference between BUP/SAM and placebo.

Analysis was performed on stage 1 and stage 2 using data from a modified intent-to-treat population defined as subjects who were randomized and received ≥1 dose of study drug (BUP/SAM or placebo) and had ≥ 1 post-baseline assessment in the relevant stage.

The evaluation of efficacy utilized mixed models for repeated measures (MMRM) to assess change from baseline at each timepoint for all treatment arms (least squares mean [LSM] estimates) as well as the BUP/SAM versus placebo difference (LSM difference [LSMD] estimates). As specified, some endpoints were based on estimates at a single timepoint and some on the average of estimates from multiple timepoints. Models included fixed effect variables for treatment group; visit; treatment group-by-visit interaction; site region, and site region-by-treatment interaction as categorical fixed effects; baseline value, and baseline-by-visit interaction as covariates. Random effects associated with patient were included as part of the marginal covariance matrix (specified as unstructured) as recommended for longitudinal data with continuous outcomes.^1^ For the pooled analysis of FORWARD-4 and FORWARD-5, an additional covariate for study was included. An unstructured covariance matrix was used to model the covariance of within-patient scores and the Kenward–Roger approximation to adjust the denominator degrees of freedom.

The primary analyses were based on combined-stage analysis where stage-specific MMRM estimates (i.e., BUP/SAM vs placebo LSMD and associated standard error) from single timepoints or the average across multiple timepoints were combined as an average using equal stage 1/stage 2 weights as defined in the statistical analysis plan (SAP). The *P*-value was obtained by referring the weighted test statistic to the standard normal distribution under the null hypothesis of no treatment effect in both stages. The proc mixed procedure in SAS v9.4 (SAS Institute, Inc., Cary, NC) was used to conduct these efficacy analyses on longitudinal data. Methods for analysis were specified in the SAP.

The primary endpoint in FORWARD-4 was the change of the MADRS-10 score from baseline to the end of the efficacy period (week 5 in both stage 1 and 2). In FORWARD-5, the first two primary endpoints used the average change from baseline to week 3 through the end-of-treatment (EOT) in MADRS-6 and MADRS-10. Therefore, for stage 1, the estimate was the average of three timepoints (the average of the change from baseline to week 3, change from baseline to week 4, and change from baseline to week 5). The estimate for stage 2 was the average of four timepoints (the average of the change from baseline to week 3, change from baseline to week 4, change from baseline to week 5, and change from baseline to week 6). For the third endpoint, the estimate for stage 1 was the change from baseline to the end of week 5 and the estimate for stage 2 was the change from baseline to the end of week 6.

Family-wise error rate (FWER) was controlled in the FORWARD-4 and FORWARD-5 studies for the primary analysis of efficacy by using a fixed sequence stepwise multiple testing procedure where the hypotheses to be tested and the order of testing were pre-specified in the SAP finalized prior to database lock. The first hypothesis in the pre-specified sequence was tested at an alpha of 0.05 (two-sided test). Each subsequent hypothesis was tested at an alpha of 0.05 (two-sided test) and could only be evaluated if the preceding null hypothesis had been rejected. All hypothesis tests were based on combined stage analysis.

The two hypotheses to be tested and their order of testing in the FORWARD-4 study were as follows: BUP/SAM 2 mg/2 mg versus placebo followed by BUP/SAM 0.5 mg/0.5 mg versus placebo for MADRS-10 score change from baseline to week 5. The six hypotheses to be tested and their order of testing in the FORWARD-4 study were as follows: BUP/SAM 2 mg/2 mg versus placebo for (1) MADRS-6 score average change from baseline to week 3 through end of treatment; (2) MADRS-10 score average change from baseline to week 3 through end of treatment; (3) MADRS-10 score change from baseline to end of treatment; and (4–6) same as hypotheses 1–3 tested for BUP/SAM 1 mg/1 mg versus placebo.

In *post-hoc* analyses, effect sizes (Hedges’ *g*) were calculated for each stage of the sequential parallel-comparison design trial and for the stages combined using Equations 1 and 2, respectively

Equation 1: $Hedge^{'}s g= \frac{M1-M2}{{s.d.}_{pooled}}$

Equation 2: $Overall Hedge^{'}s g= \frac{w\hat{\theta}^{(1)}+(1-w)\hat{\theta}^{(2)}}{\sqrt{w^{2}Var(\hat{\theta}^{\left( 1 \right)}+\left( 1-w \right)^{2}Var(\hat{\theta}^{\left( 2 \right)})}}$

Where M1 and M2 are the mean reductions from baseline of active and placebo groups, respectively, s.d._pooled_ is the pooled standard deviation calculated from the model standard error, w = 0.5, and $\hat{\theta}^{\left( 1 \right)}$ and $\hat{\theta}^{\left( 2 \right)}$ refer to the effect sizes for the two stages. Equation 2 assumes the effect sizes are asymptotically independent between the two stages and the combined-stage effect size is the average of the stage-specific effect sizes.

As the FORWARD-4 and FORWARD-5 studies employed nearly identical designs, a pooled analysis was performed to improve precision and accuracy of the results with the increased sample size using the statistical methods described above. The analyses based on average change from baseline estimated from multiple timepoints use more data than analyses based on change from baseline to a single timepoint. As such, these estimates have smaller standard error (making them more precise) and reflect a more stable estimate (making them more accurate) that is not susceptible to visit-to-visit fluctuations. The three primary endpoints and adjustment for multiple comparisons were identical to those described for FORWARD-5 and pre-specified before unblinding of FORWARD-5, but after unblinding of FORWARD-4.

## References

1. Mallinckrodt CH, Lane PW, Schnell D, Peng Y, Mancuso, JM. Recommendations for the primary analysis of continuous endpoints in longitudinal clinical trials. *Drug Inf J*. 2008; 42: 303-319.

**Supplementary Table 1.** MADRS-10 treatment response and remission rates at EOT (LOCF) in FORWARD-4, FORWARD-5, and the pooled analysis.

|  | *FORWARD-4* | | | | *FORWARD-5* | | | | *Pooled* | | | |
| --- | --- | --- | --- | --- | --- | --- | --- | --- | --- | --- | --- | --- |
|  | *Stage 1, week 5* | | *Stage 2, week 5* | | *Stage 1, week 5* | | *Stage 2, week 6* | | *Stage 1, week 5* | | *Stage 2, week 6* | |
|  | *PBO + ADT*  *(*N *= 256)* | *BUP/SAM (2 mg/ 2 mg) + ADT*  *(*N *= 59)* | *PBO + ADT*  *(*N *= 54)* | *BUP/SAM (2 mg/ 2 mg) + ADT*  *(*N *= 54)* | *PBO + ADT*  *(*N *= 273)* | *BUP/SAM (2 mg/ 2 mg) + ADT*  *(*N *= 63)* | *PBO + ADT*  *(*N *= 60)* | *BUP/SAM (2 mg/ 2 mg) + ADT*  *(*N *= 63)* | *PBO + ADT*  *(*N *= 529)* | *BUP/SAM (2 mg/ 2 mg) + ADT*  *(*N *= 122)* | *PBO + ADT (*N*=114)* | *BUP/SAM (2 mg/ 2 mg) + ADT*  *(*N *= 117)* |
| *Response rate* | | | | | | | | | | | | |
| *n* (%) | 79 (30.9) | 20 (33.9) | 6 (11.1) | 9 (16.7) | 61 (22.3) | 16 (25.4) | 7 (11.7) | 6 (9.5) | 140 (26.5) | 36 (29.5) | 12 (10.5) | 14 (12.0) |
| Weighted difference versus PBO, rate (s.e.), *P*-value | 4.3 (4.8)  *P*-value = 0.366 | | | | 0.5 (4.1)  *P*-value = 0.912 | | | | 2.2 (3.1)  *P*-value = 0.467 | | | |
| *Remission rate* | | | | | | | | | | | | |
| *n* (%) | 47(18.4) | 13 (22.0) | 4 (7.4) | 7 (13.0) | 31 (11.4) | 8 (12.7) | 4 (6.7) | 5 (7.9) | 78 (14.7) | 21 (17.2) | 8 (7.0) | 13 (11.1) |
| Weighted differenced versus PBO, rate (s.e.),  *P*-value | 4.6 (4.1)  *P*-value = 0.265 | | | | 1.3 (3.3)  *P*-value = 0.691 | | | | 3.3 (2.7)  *P*-value = 0.217 | | | |
| Abbreviations: ADT, antidepressant therapy; BUP, buprenorphine; EOT, end-of-treatment; LOCF, last observation carried forward; MADRS, Montgomery–Åsberg Depression Rating Scale; PBO, placebo; SAM, samidorphan; s.e., standard error.  MADRS treatment response was defined as a ≥50% reduction in MADRS total score from baseline. | | | | | | | | | | | | |

**Supplementary Table 2.** Summary of effect size of BUP/SAM (2 mg/2 mg) + ADT in FORWARD-4, FORWARD-5, and the pooled analysis.

| Hedges’ g (95% CI) | FORWARD-4 | | | FORWARD-5 | | | Pooled | | |
| --- | --- | --- | --- | --- | --- | --- | --- | --- | --- |
|  | Stage 1 | Stage 2 | Overall | Stage 1 | Stage 2 | Overall | Stage 1 | Stage 2 | Overall |
| MADRS-6 average change in baseline from week 3 to EOT | 0.21(–0.07, 0.49) | 0.54(0.16, 0.93) | 0.38(0.14, 0.62) | 0.22(–0.05, 0.50) | 0.32(–0.04, 0.68) | 0.27(0.05, 0.50) | 0.22(0.03, 0.42) | 0.35(0.09, 0.61) | 0.29(0.12, 0.45) |
| MADRS-10 average change in baseline from week 3 to EOT | 0.21(–0.07, 0.50) | 0.35(–0.03, 0.73) | 0.28(0.05, 0.52) | 0.27(–0.01, 0.54) | 0.23(–0.12, 0.59) | 0.25(0.03, 0.48) | 0.24(0.04, 0.43) | 0.23(–0.03, 0.49) | 0.23(0.07, 0.40) |
| MADRS-10 change from baseline to EOT | 0.17(–0.11, 0.46) | 0.40(0.01, 0.78) | 0.28(0.05, 0.52) | 0.18(–0.10, 0.45) | 0.23(–0.13, 0.58 | 0.20(–0.02, 0.43) | 0.17(–0.02, 0.37) | 0.26(0.00, 0.52) | 0.22(0.05, 0.38) |
| Abbreviations: ADT, antidepressant therapy; BUP, buprenorphine; CI, confidence interval; EOT, end-of-treatment; MADRS, Montgomery–Åsberg Depression Rating Scale; SAM, samidorphan. | | | | | | | | | |

**Supplementary Table 3.** Safety events among patients in FORWARD-4 and FORWARD-5.

| *Event,* n *(%)* | *FORWARD-4* | | | | | | *FORWARD-5* | | | | | |
| --- | --- | --- | --- | --- | --- | --- | --- | --- | --- | --- | --- | --- |
|  | *Stage 1* | | | *Stage 2* | | | *Stage 1* | | | *Stage 2* | | |
|  | *PBO  + ADT* | *BUP/SAM + ADT* | | *PBO  + ADT* | *BUP/SAM + ADT* | | *PBO  + ADT* | *BUP/SAM + ADT* | | *PBO  + ADT* | *BUP/SAM + ADT* | |
|  | *(*n *= 265)* | *0.5 mg/*  *0.5 mg (*n *= 59)* | *2 mg/*  *2 mg (*n *= 60)* | *(*n *= 56)* | *0.5 mg/*  *0.5 mg (*n *= 56)* | *2 mg/*  *2 mg (*n *= 56)* | *(*n *= 280)* | *1 mg/*  *1 mg (*n *= 63)* | *2 mg/*  *2 mg (*n *= 63)* | *(*n *= 62)* | *1mg/*  *1 mg (*n *= 62)* | *2 mg/*  *2 mg (*n *= 63)* |
| *Any AE* | 142 (53.6) | 34 (57.6) | 41 (68.3) | 29 (51.8) | 27 (48.2) | 29 (51.8) | 151 (53.9) | 37 (58.7) | 42 (66.7) | 25 (40.3) | 29 (46.8) | 25 (39.7) |
| *Any SAE* | 1 (0.4) | 0 | 0 | 0 | 0 | 0 | 1 (0.4) | 0 | 2 (3.2) | 1 (1.6) | 0 | 0 |
| *AE leading to study discontinuation* | 6 (2.3) | 4 (6.8) | 7 (11.7) | 0 | 0 | 1 (1.8) | 6 (2.1) | 5 (7.9) | 11 (17.5) | 2 (3.2) | 3 (4.8) | 3 (4.8) |
| *Common AEs^a^* | | | | | | | | | | | | |
| Nausea | 17 (6.4) | 14 (23.7) | 17 (28.3) | 1 (1.8) | 5 (8.9) | 8 (14.3) | 20 (7.1) | 9 (14.3) | 17 (27.0) | 1 (1.6) | 2 (3.2) | 5 (7.9) |
| Constipation | 4 (1.5) | 4 (6.8) | 10 (16.7) | 1 (1.8) | 1 (1.8) | 2 (3.6) | 9 (3.2) | 9 (14.3) | 5 (7.9) | 0 | 2 (3.2) | 4 (6.3) |
| Dizziness | 9 (3.4) | 4 (6.8) | 8 (13.3) | 1 (1.8) | 4 (7.1) | 2 (3.6) | 12 (4.3) | 6 (9.5) | 7 (11.1) | 1 (1.6) | 1 (1.6) | 2 (3.2) |
| Somnolence | 7 (2.6) | 5 (8.5) | 6 (10.0) | 0 | 1 (1.8) | 0 | 12 (4.3) | 4 (6.3) | 3 (4.8) | 0 | 0 | 0 |
| Vomiting | 4 (1.5) | 4 (6.8) | 6 (10.0) | 0 | 2 (3.6) | 4 (7.1) | 7 (2.5) | 3 (4.8) | 6 (9.5) | 1 (1.6) | 0 | 1 (1.6) |
| Dry mouth | 11 (4.2) | 2 (3.4) | 5 (8.3) | 0 | 0 | 2 (3.6) | 11 (3.9) | 2 (3.2) | 1 (1.6) | 0 | 1 (1.6) | 1 (1.6) |
| Headache | 22 (8.3) | 7 (11.9) | 5 (8.3) | 1 (1.8) | 1 (1.8) | 2 (3.6) | 22 (7.9) | 4 (6.3) | 5 (7.9) | 4 (6.5) | 0 | 2 (3.2) |
| Insomnia | 7 (2.6) | 1 (1.7) | 5 (8.3) | 1 (1.8) | 0 | 0 | 3 (1.1) | 2 (3.2) | 1 (1.6) | 1 (1.6) | 1 (1.6) | 0 |
| Sedation | 3 (1.1) | 2 (3.4) | 5 (8.3) | 0 | 0 | 0 | 1 (0.4) | 3 (4.8) | 3 (4.8) | 0 | 0 | 0 |
| Hyperhidrosis | 4 (1.5) | 0 | 4 (6.7) | 0 | 0 | 1 (1.8) | 6 (2.1) | 3 (4.8) | 3 (4.8) | 0 | 0 | 1 (1.6) |
| Abnormal dreams | 7 (2.6) | 3 (5.1) | 3 (5.0) | 0 | 0 | 0 | 4 (1.4) | 2 (3.2) | 2 (3.2) | 0 | 1 (1.6) | 0 |
| Hot flush | 5 (1.9) | 0 | 3 (5.0) | 0 | 0 | 1 (1.8) | 2 (0.7) | 0 | 0 | 0 | 0 | 0 |
| Irritability | 0 | 0 | 3 (5.0) | 0 | 0 | 1 (1.8) | 1 (0.4) | 1 (1.6) | 0 | 0 | 1 (1.6) | 0 |
| Muscle spasms | 1 (0.4) | 0 | 3 (5.0) | 1 (1.8) | 0 | 0 | 0 | 0 | 0 | 0 | 1 (1.6) | 0 |
| Fatigue | 5 (1.9) | 3 (5.1) | 2 (3.3) | 1 (1.8) | 0 | 3 (5.4) | 1 (0.4) | 5 (7.9) | 7 (11.1) | 1 (1.6) | 0 | 1 (1.6) |
| Anxiety | 3 (1.1) | 0 | 2 (3.3) | 0 | 0 | 0 | 2 (0.7) | 1 (1.6) | 1 (1.6) | 2 (3.2) | 1 (1.6) | 0 |
| Decreased appetite | 2 (0.8) | 0 | 2 (3.3) | 0 | 0 | 1 (1.8) | 3 (1.1) | 1 (1.6) | 3 (4.8) | 0 | 1 (1.6) | 0 |
| Hematuria | 0 | 0 | 2 (3.3) | 0 | 0 | 0 | 1 (0.4) | 0 | 0 | 0 | 0 | 0 |
| Oropharyngeal pain | 1 (0.4) | 0 | 2 (3.3) | 2 (3.6) | 0 | 1 (1.8) | 1 (0.4) | 0 | 0 | 0 | 0 | 0 |
| Pruritus | 1 (0.4) | 2 (3.4) | 2 (3.3) | 1 (1.8) | 1 (1.8) | 0 | 3 (1.1) | 0 | 1 (1.6) | 0 | 0 | 1 (1.6) |
| Thirst | 0 | 0 | 2 (3.3) | 0 | 0 | 0 | 1 (0.4) | 1 (1.6) | 0 | 0 | 0 | 0 |
| Upper RTI | 6 (2.3) | 1 (1.7) | 2 (3.3) | 4 (7.1) | 0 | 2 (3.6) | 4 (1.4) | 1 (1.6) | 1 (1.6) | 2 (3.2) | 3 (4.8) | 0 |
| Abdominal discomfort | 3 (1.1) | 2 (3.4) | 1 (1.7) | 0 | 0 | 0 | 1 (0.4) | 0 | 0 | 0 | 0 | 0 |
| Diarrhea | 7 (2.6) | 2 (3.4) | 1 (1.7) | 1 (1.8) | 1 (1.8) | 1 (1.8) | 11 (3.9) | 0 | 3 (4.8) | 0 | 2 (3.2) | 0 |
| Nasopharyngitis | 6 (2.3) | 1 (1.7) | 0 | 2 (3.6) | 1 (1.8) | 3 (5.4) | 8 (2.9) | 1 (1.6) | 3 (4.8) | 4 (6.5) | 2 (3.2) | 1 (1.6) |
| Arthralgia | 3 (1.1) | 0 | 0 | 0 | 0 | 2 (3.6) | 1 (0.4) | 2 (3.2) | 0 | 0 | 0 | 1 (1.6) |
| Back pain | 6 (2.3) | 1 (1.7) | 0 | 1 (1.8) | 2 (3.6) | 2 (3.6) | 4 (1.4) | 0 | 0 | 0 | 1 (1.6) | 0 |
| Bronchitis | 2 (0.8) | 0 | 1 (1.7) | 1 (1.8) | 0 | 2 (3.6) | 2 (0.7) | 0 | 0 | 0 | 0 | 0 |
| Dyspepsia | 1 (0.4) | 0 | 1 (1.7) | 0 | 1 (1.8) | 2 (3.6) | 2 (0.7) | 0 | 2 (3.2) | 0 | 0 | 0 |
| Gastroenteritis | 2 (0.8) | 0 | 1 (1.7) | 0 | 1 (1.8) | 2 (3.6) | 1 (0.4) | 0 | 1 (1.6) | 0 | 0 | 0 |
| Laceration | 0 | 0 | 0 | 0 | 2 (3.6) | 0 | 0 | 0 | 0 | 0 | 0 | 0 |
| Flatulence | 0 | 0 | 0 | 2 (3.6) | 0 | 1 | 2 (0.7) | 0 | 2 (3.2) | 0 | 0 | 0 |
| Paresthesia | 0 | 0 | 0 | 0 | 0 | 1 (1.8) | 0 | 0 | 2 (3.2) | 0 | 0 | 0 |
| Sleep disorder | 1 (0.4) | 0 | 0 | 0 | 0 | 0 | 1 (0.4) | 0 | 2 (3.2) | 0 | 0 | 0 |
| Myalgia | 3 (1.1) | 0 | 0 | 0 | 0 | 0 | 0 | 2 (3.2) | 0 | 1 (1.6) | 0 | 0 |
| UTI | 1 (0.4) | 0 | 1 (1.7) | 1 (1.8) | 1 (1.8) | 1 (1.8) | 2 (0.7) | 1 (1.6) | 1 (1.6) | 1 (1.6) | 3 (4.8) | 1 (1.6) |
| *Any AESI of abuse potential that is euphoria related* | | | | | | | | | | | | |
| Feeling abnormal | 1 (0.4) | 0 | 1 (1.7) | 0 | 0 | 0 | 0 | 1 (1.6) | 1 (1.6) | 1 (0.8) | 0 | 0 |
| Euphoric mood | 0 | 0 | 1 (1.7) | 0 | 0 | 0 | 0 | 0 | 0 | 0 | 0 | 0 |
| Feeling of relaxation | 0 | 0 | 1 (1.7) | 0 | 0 | 0 | 0 | 0 | 0 | 0 | 0 | 0 |
| *Any AESI of abuse potential that is non-specific* | | | | | | | | | | | | |
| Dizziness | 9 (3.4) | 4 (6.8) | 8 (13.3) | 1 (1.8) | 4 (7.1) | 2 (3.6) | 12 (4.3) | 6 (9.5) | 7 (11.1) | 1 (1.6) | 1 (1.6) | 2 (3.2) |
| Somnolence | 7 (2.6) | 5 (8.5) | 6 (10.0) | 0 | 1 (1.8) | 0 | 12 (4.3) | 4 (6.3) | 3 (4.8) | 0 | 0 | 0 |
| Sedation | 3 (1.1) | 2 (3.4) | 5 (8.3) | 0 | 0 | 0 | 1 (0.4) | 3 (4.8) | 3 (4.8) | 0 | 0 | 0 |
| Emotional disorder | 1 (0.4) | 0 | 0 | 0 | 0 | 0 | 0 | 0 | 0 | 0 | 0 | 0 |
| Abbreviations: ADT, antidepressant therapy; AE, adverse event; BUP, buprenorphine; PBO, placebo; RTI, respiratory tract infection; SAE, serious adverse event; SAM, samidorphan; UTI, urinary tract infection.  ^a^Occurring in ≥2% of patients in any of the BUP/SAM treatment groups. | | | | | | | | | | | | |

**Supplementary Table 4.** Postdiscontinuation-emergent AEs associated with withdrawal in the pooled analysis.

|  | *Pooled* | |
| --- | --- | --- |
|  | PBO + ADT *(*N *= 165)* | *BUP/SAM (2 mg/2 mg) + ADT  (*N *= 132)* |
| *Any postdiscontinuation event associated with withdrawal, mean (s.d.)* | 4 (2.4) | 4 (3.0) |
| Insomnia | 1 (0.6) | 1 (0.8) |
| Anxiety | 0 | 0 |
| Chills | 0 | 1 (0.8) |
| Drug withdrawal syndrome | 0 | 1 (0.8) |
| Hyperhidrosis | 1 (0.6) | 1 (0.8) |
| Irritability | 1 (0.6) | 0 |
| Rhinorrhea | 1 (0.6) | 0 |
| Tremor | 1 (0.6) | 0 |
| Abbreviations: ADT, antidepressant therapy; AE, adverse event; BUP, buprenorphine; PBO, placebo; SAM, samidorphan; s.d., standard deviation. | | |

**Supplementary Table 5.** Summary of COWS scores in FORWARD-4, FORWARD-5, and the pooled analysis.

|  | | FORWARD-4 | | | FORWARD-5 | | | Pooled | |
| --- | --- | --- | --- | --- | --- | --- | --- | --- | --- |
|  |  | PBO  + ADT(N = 153) | BUP/SAM (0.5 mg/0.5 mg)+ ADT (N = 115) | BUP/SAM(2 mg/2 mg) + ADT (N = 116) | PBO  + ADT(N = 155) | BUP/SAM (0.5 mg/0.5 mg)  + ADT (N = 125) | BUP/SAM(2 mg/2 mg) + ADT (N = 126) | PBO + ADT(N = 146) | BUP/SAM(2 mg/2 mg) + ADT  (N = 107) |
| COWS score at EOT or early termination | n | 145 | 107 | 111 | 42 | 41 | 38 | 146 | 107 |
|  | Mean (s.d.) | 0.61 (1.1) | 0.81 (1.6) | 0.75 (1.1) | 0.69 (1.3) | 0.78 (1.2) | 1.45 (1.8) | 0.7 (1.3) | 0.6 (1.1) |
| Change in COWS score from final treatment visit to postdiscontinuation visit | n | 141 | 104 | 110 | 19 | 22 | 18 | 146 | 107 |
|  | Mean (s.d.) | 0.09 (1.4) | –0.02 (1.2) | 0.14 (2.0) | 0.11 (1.6) | –0.32 (1.3) | –0.33 (1.9) | 0.1 (1.4) | 0.3 (2.0) |
| COWS score category postdiscontinuation,n (%) | N | 141 | 104 | 110 | 19 | 23 | 19 | 146 | 107 |
|  | No withdrawal | 137 (97.2) | 99 (95.2) | 105 (95.5) | 19 (100.0) | 19 (100.0) | 19 (100.0) | 142 (97.3) | 102 (95.3) |
|  | Mild withdrawal | 4 (2.8) | 5 (4.8) | 4 (3.6) | 2 (4.8) | 1 (2.4) | 4 (10.5) | 4 (2.7) | 4 (3.7) |
|  | Moderate withdrawal | 0 | 0 | 1 (0.9) | 0 | 0 | 0 | 0 | 1 (0.9) |
|  | Moderately severe | 0 | 0 | 0 | 0 | 0 | 0 | 0 | 0 |
|  | Severe withdrawal | 0 | 0 | 0 | 0 | 0 | 0 | 0 | 0 |
| Abbreviations: ADT, antidepressant therapy; BUP, buprenorphine; COWS, Clinical Opiate Withdrawal Scale; EOT, end-of-treatment; PBO, placebo; SAM, samidorphan; s.d., standard deviation. | | | | | | | | | |

**Supplementary Table 6.** Summary of suicidal behavior and ideation during stage 1 and 2 in FORWARD-4 and FORWARD-5 (C-SSRS)

| *Category,*  n *(%)* | *FORWARD-4* | | | | | | *FORWARD-5* | | | | | | | | | | | *Pooled* | | | | | | |
| --- | --- | --- | --- | --- | --- | --- | --- | --- | --- | --- | --- | --- | --- | --- | --- | --- | --- | --- | --- | --- | --- | --- | --- | --- |
|  | *Stage 1* | | | *Stage 2* | | | *Stage 1* | | | | | *Stage 2* | | | | | | *Stage 1* | | | | *Stage 2* | | |
|  | *PBO  + ADT*  *(*N *= 265)* | *BUP/*  *SAM*  *(0.5 mg/*  *0.5 mg) + ADT*  *(*N *= 59)* | *BUP/*  *SAM*  *(2 mg/*  *2 mg)  + ADT*  *(*N *= 60)* | *PBO  + ADT*  *(*N *= 56)* | *BUP/*  *SAM*  *(0.5 mg/*  *0.5 mg) + ADT*  *(*N *= 56)* | *BUP/*  *SAM*  *(2 mg/*  *2 mg)  + ADT*  *(*N *= 56)* | *PBO + ADT*  *(*N *= 280)* | *BUP/*  *SAM*  *(1 mg/*  *1 mg)  + ADT*  *(*N *= 63)* | | *BUP/*  *SAM*  *(2 mg/*  *2 mg)  + ADT*  *(*N *= 63)* | | *PBO  + ADT*  *(*N *= 62)* | | *BUP/*  *SAM*  *(1 mg/*  *1 mg)  + ADT*  *(*N *= 62)* | | *BUP/*  *SAM*  *(2 mg/*  *2 mg)  + ADT*  *(N = 63)* | | *PBO  + ADT*  *(*N *= 545)* | | *BUP/*  *SAM*  *(2 mg/*  *2 mg)  + ADT*  *(*N *= 123)* | | *PBO  + ADT*  *(*N *= 118)* | | *BUP/*  *SAM*  *(2 mg/*  *2 mg)  + ADT (*N *= 119)* |
| *Baseline* | | | | | | | | | | | | | | | | | | | | | | | | |
| *Suicidal ideation* | 39 (14.7) | 4 (6.8) | 13 (21.7) | 9 (16.1) | 9 (16.1) | 6 (10.7) | 26 (9.3) | 12 (19.0) | 9 (14.3) | | 1 (1.6) | | 4 (6.5) | | 4 (6.3) | | 65 (11.9) | | 22 (17.9) | | 10 (8.5) | | 10 (8.4) | |
| *Suicidal behavior* | 0 | 0 | 0 | 0 | 0 | 0 | 0 | 0 | 0 | | 0 | | 0 | | 0 | | 0 | | 0 | | 0 | | 0 | |
| *Nonsuicidal self-injurious behavior* | 1 (0.4) | 0 | 0 | 0 | 1 (1.8) | 0 | 0 | 0 | 0 | | 0 | | 0 | | 0 | | 1 (0.2) | | 0 | | 0 | | 0 | |
| *Any postbaseline visit* | | | | | | | | | | | | | | | | | | | | | | | | |
| *Suicidal ideation* | 54 (20.4) | 9 (15.3) | 8 (13.3) | 13 (23.2) | 12 (21.4) | 4 (7.1) | 31 (11.1) | 12 (19.0) | 5 (7.9) | | 6 (9.7) | | 7 (11.3) | | 5 (7.9) | | 85 (15.6) | | 13 (10.6) | | 19 (6.1) | | 9 (7.6) | |
| *Wish to be dead* | 53 (20.0) | 8 (13.6) | 8 (13.3) | 13 (23.2) | 12 (21.4) | 4 (7.1) | 31 (11.1) | 12 (19.0) | 5 (7.9) | | 6 (9.7) | | 7 (11.3) | | 5 (7.9) | | 84 (15.4) | | 13 (10.6) | | 19 (6.1) | | 9 (7.6) | |
| *Nonspecific active suicidal thoughts* | 14 (5.3) | 3 (5.1) | 3 (5.0) | 4 (7.1) | 1 (1.8) | 1 (1.8) | 5 (1.8) | 4 (6.3) | 0 | | 0 | | 1 (1.6) | | 1 (1.6) | | 19 (3.5) | | 3 (2.4) | | 4 (3.4) | | 2 (1.7) | |
| *Active ideation without intent to act* | 9 (3.4) | 0 | 0 | 3 (5.4) | 1 (1.8) | 0 | 2 (0.7) | 2 (3.2) | 0 | | 0 | | 1 (1.6) | | 1 (1.6) | | 11 (2.0) | | 0 | | 3 (2.5) | | 1 (0.8) | |
| *Active ideation with some intent to act without a plan* | 1 (0.4) | 0 | 0 | 0 | 0 | 0 | 1 (0.4) | 0 | 0 | | 0 | | 0 | | 0 | | 2 (0.4) | | 0 | | 0 | | 0 | |
| *Active ideation with a specific plan and intent* | 0 | 0 | 0 | 0 | 0 | 0 | 0 | 0 | 0 | | 0 | | 0 | | 0 | | 0 | | 0 | | 0 | | 0 | |
| *Suicidal behavior* | 1 (0.4) | 0 | 0 | 0 | 0 | 0 | 1 (0.4) | 0 | 0 | | 1 (1.6) | | 0 | | 0 | | 2 (0.4) | | 0 | | 1 (0.8) | | 0 | |
| *Actual attempt* | 0 | 0 | 0 | 0 | 0 | 0 | 0 | 0 | 0 | | 1 (1.6) | | 0 | | 0 | | 0 | | 0 | | 1 (0.8) | | 0 | |
| *Interrupted attempt* | 0 | 0 | 0 | 0 | 0 | 0 | 0 | 0 | 0 | | 1 (1.6) | | 0 | | 0 | | 0 | | 0 | | 1 (0.8) | | 0 | |
| *Preparatory acts or behavior* | 1 (0.4) | 0 | 0 | 0 | 0 | 0 | 1 (0.4) | 0 | 0 | | 0 | | 0 | | 0 | | 2 (0.4) | | 0 | | 0 | | 0 | |
| *Nonsuicidal self-injurious behavior* | 1 (0.4) | 0 | 0 | 0 | 1 (1.8) | 0 | 1 (0.4) | 0 | 0 | | 0 | | 0 | | 0 | | 2 (0.4) | | 0 | | 0 | | 0 | |
| Abbreviations: ADT, antidepressant therapy; BUP, buprenorphine; C-SSRS, Columbia Suicide Severity Rating Scale; PBO, placebo; SAM, samidorphan. | | | | | | | | | | | | | | | | | | | | | | | | |

**Supplementary Figure 1A.** FORWARD-4 patient disposition.


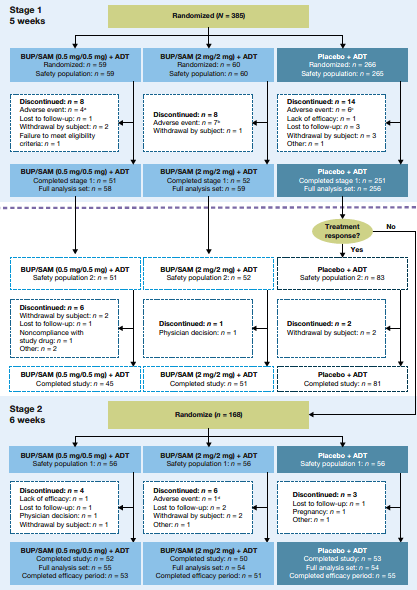


^a^AEs leading to study discontinuation in the BUP/SAM 0.5 mg/0.5 mg group during stage 1 were anemia, vomiting, diarrhea, fatigue, somnolence, and headache. ^b^AEs leading to study discontinuation in the BUP/SAM 2 mg/2 mg group during stage 1 were nausea, abdominal discomfort, dry mouth, vomiting, diarrhea, dizziness, somnolence, insomnia, irritability, hyperhidrosis, and pruritus. ^c^AEs leading to study discontinuation in the placebo group during stage 1 were pharyngitis streptococcal, musculoskeletal stiffness, somnolence, insomnia, anxiety, and depression. ^d^AE leading to study discontinuation in the BUP/SAM 2 mg/2 mg group during stage 2 was presyncope.

ADT, antidepressant therapy; AE, adverse event; BUP, buprenorphine; SAM, samidorphan.

**Supplementary Figure 1B.** FORWARD-5 patient disposition.


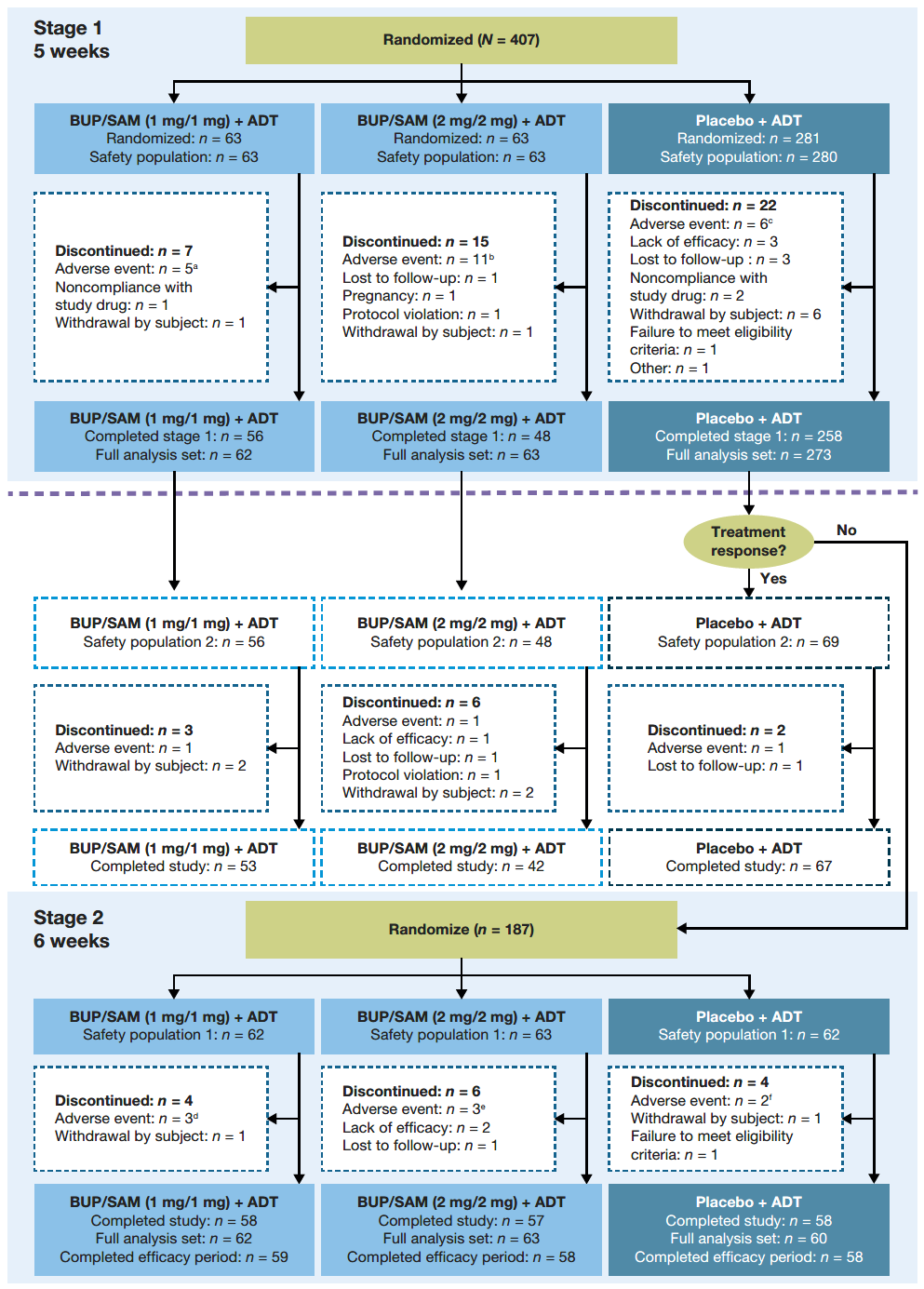


^a^AEs leading to study discontinuation in the BUP/SAM 1 mg/1 mg group during stage 1 were nausea, fatigue, headache, and emotional disorder. ^b^AEs leading to study discontinuation in the BUP/SAM 2 mg/2 mg group during stage 1 were visual impairment, nausea, vomiting, abdominal pain, constipation, dyspepsia, asthenia, feeling abnormal, muscle strain, muscle twitching, dizziness, headache, sedation, depression, and nervousness. ^c^AEs leading to study discontinuation in the placebo group during stage 1 were extrasystoles, nausea, hypersensitivity, headache, somnolence, and insomnia. ^d^AEs leading to study discontinuation in the BUP/SAM 1 mg/1 mg group during stage 2 were extrasystoles, alanine aminotransferase increased, aspartate aminotransferase increased, blood alkaline phosphatase increased, blood lactate dehydrogenase increased, γ-glutamyl transferase increased, liver function test abnormal, and neck pain. ^e^AEs leading to study discontinuation in the BUP/SAM 2 mg/2 mg group during stage 2 were nausea, vomiting, dizziness, and derealization. ^f^AEs leading to study discontinuation in the placebo group during stage 2 were liver function test abnormal, depression, and suicide attempt.

ADT, antidepressant therapy; AE, adverse event; BUP, buprenorphine; SAM, samidorphan.

**Supplementary Figure 2.** BUP/SAM (2 mg/2 mg) + ADT LSMD from placebo + ADT in the change from baseline in MADRS-6 scores across the entire study in (A) FORWARD-4, (B) FORWARD-5, and (C) the pooled analysis. Shading indicates primary endpoints for each study.


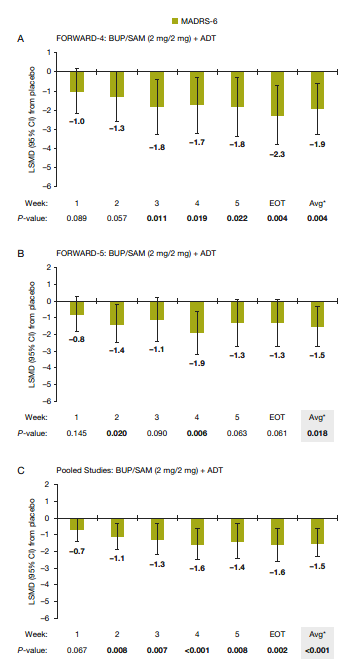


**Supplementary Figure 3.** LSMD from placebo in the change from baseline in MADRS scores across the entire study for (A) BUP/SAM (0.5 mg/0.5 mg) + ADT in FORWARD-4 and (B) BUP/SAM (1 mg/1 mg) + ADT in FORWARD-5. Shading indicates primary endpoints for each study.


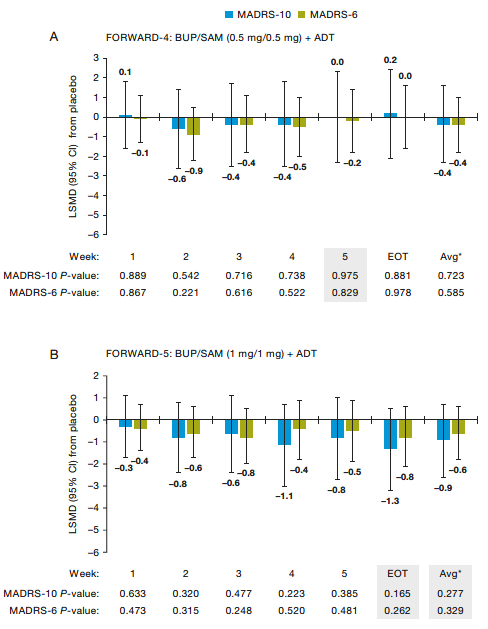


**Supplementary Figure 4.** Mean change from baseline MADRS-10 scores by visit during the entire treatment period for the BUP/SAM (2 mg/2 mg) + ADT and placebo + ADT treatment groups in (A) FORWARD-4 and (B) FORWARD-5 Stage 1 full analysis sets


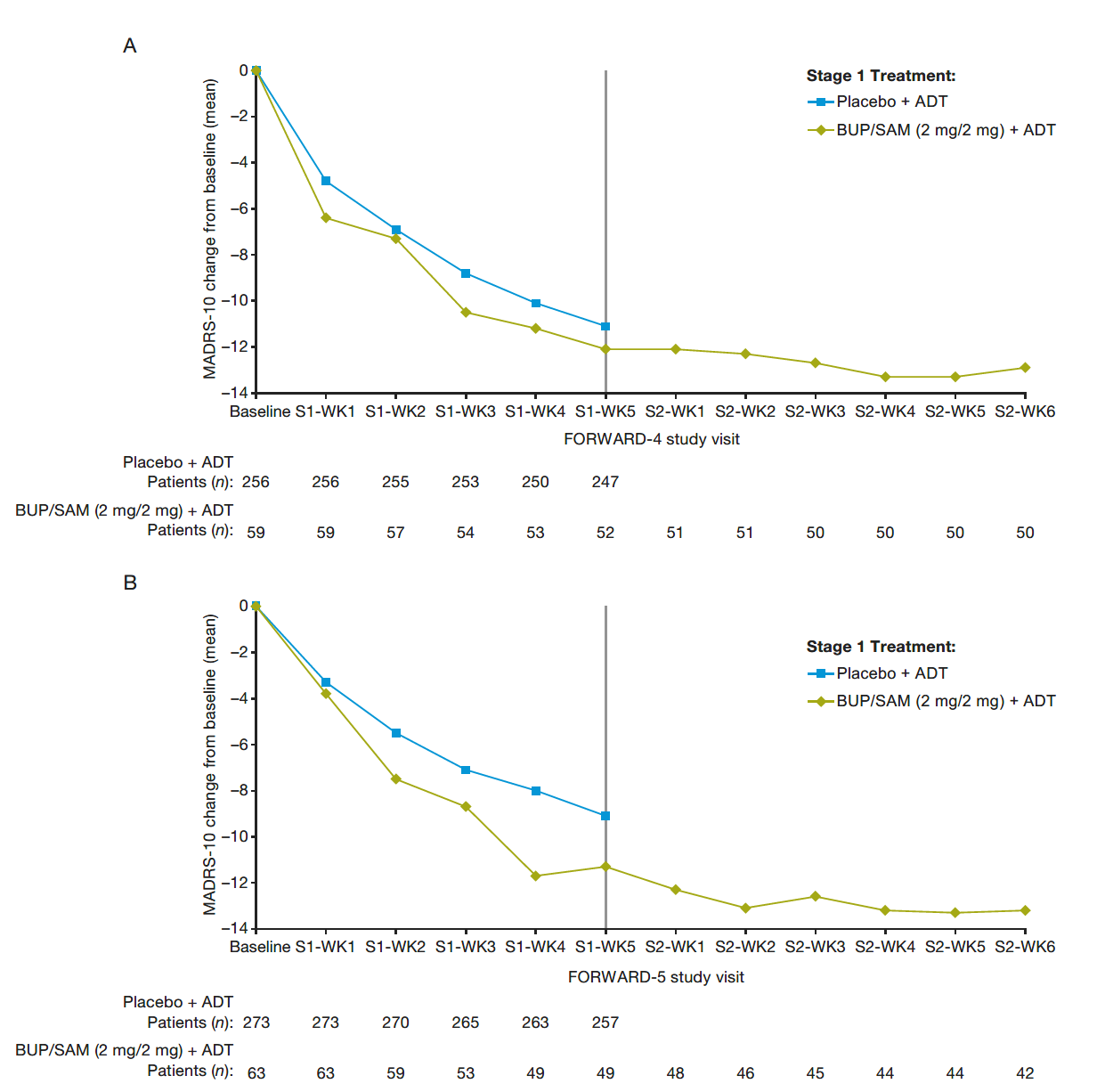


ADT, antidepressant therapy; BUP, buprenorphine; MADRS, Montgomery–Åsberg Depression Rating Scale; S, stage; SAM, samidorphan; WK, week.
